# 3‐D Sustained‐Release Culture Carrier Alleviates Rat Intervertebral Disc Degeneration by Targeting STING in Transplanted Skeletal Stem Cells

**DOI:** 10.1002/advs.202410151

**Published:** 2025-02-22

**Authors:** Liwen Luo, Shiyu Zhang, Junfeng Gong, Ji Zhang, Peng Xie, Jun Yin, MengJie Zhang, Cong Zhang, Hong Chen, Yao Liu, Bing Ni, Changqing Li, Zhiqiang Tian

**Affiliations:** ^1^ Department of Orthopaedics Xinqiao Hospital Army Medical University (Third Military Medical University) Chongqing P. R. China; ^2^ State Key Laboratory of Trauma and Chemical Poisoning Army Medical University (Third Military Medical University) Chongqing P. R. China; ^3^ Department of General Surgery The Armed Police Corps Hospital of Anhui Hefei P. R. China; ^4^ Institute of Immunology PLA Army Medical University (Third Military Medical University) Chongqing P. R. China; ^5^ Department of Military Biosafety College of Basic Medicine Army Medical University Chongqing P. R. China; ^6^ Department of Pathophysiology College of High Altitude Military Medicine Army Military Medical University Chongqing P. R. China; ^7^ Department of Laboratory Animal Science College of Basic Medicine Army Medical University Chongqing P. R. China; ^8^ Department of Orthopedics 903 Hospital of Joint Logistic Support Force of The People's Liberation Army Hangzhou P. R. China; ^9^ Department of Pharmacy Daping Hospital Army Medical University (Third Military Medical University) Chongqing P. R. China

**Keywords:** cGAS/STING, hypoxia, intervertebral disc degeneration, reactive oxygen species, skeletal stem cells

## Abstract

The hypoxic and high‐pressure microenvironment of the intervertebral discs poses a major challenge to the survival and therapeutic efficiency of exogenous stem cells. Therefore, improving the utilization efficiency and therapeutic effect of exogenous stem cells to delay intervertebral disc degeneration (IVDD) is of great importance. Here, hypoxic induction studies are conducted in vivo and in vitro using rat costal cartilage‐derived skeletal stem cells (SSCs) and find that hypoxia activates the cyclic guanosine monophosphate–adenosine monophosphate synthase (cGAS)/stimulator of interferon genes (STING) signaling pathway and increased reactive oxygen species (ROS) accumulation, triggering ferroptosis in SSCs through hypoxia‐inducible factor‐1 alpha‐dependent mitophagy. Progressive hypoxia preconditioning reduce STING expression and ROS accumulation, inducing SSCs differentiation into nucleus pulposus‐like cells via the Wnt signaling pathway. Considering this, a 3‐D sustained‐release culture carrier is generated by mixing SSCs with methacrylated hyaluronic acid and polydopamine nanoparticles coated with the STING inhibitor C‐176 and evaluated its inhibitory effect on IVDD. This carrier is demonstrated to inhibit the cGAS/STING pathway and prevent ROS accumulation by continuously releasing C‐176‐coated polydopamine nanoparticles, thereby reducing ferroptosis, promoting differentiation, and ultimately attenuating IVDD, suggesting its potential as a novel treatment strategy.

## Graphical abstract

Schematic representation of mechanisms by which the 3‐D sustained‐release culture carrier prevents accumulation of ROS by continuously releasing C‐176@PDA‐NPs and inhibiting the cGAS‐STING activated by sudden hypoxia, thereby preventing ferroptosis and promoting differentiation in SSCs and ultimately attenuating IVDD progression.

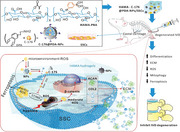

## 1 Introduction

Neck and low back disorders are the leading causes of disability worldwide, causing severe pain and seriously impairing patients’ quality of life.^[^ ^1^ ^]^ Intervertebral disc degeneration (IVDD) is a major cause of neck and low back pain.^[^ ^2^ ^]^ Although stem cells obtained from bone marrow and adipose tissues have great clinical potential for treating IVDD,^[^ ^3^, ^4^ ^]^ they are associated with potential disadvantages such as tumorigenic properties and promoting carcinoma metastasis.^[^ ^5^, ^6^, ^7^ ^]^ Recently, mouse and human skeletal stem cells (SSCs) have been identified as a possible tool to promote articular cartilage or bone regeneration due to their ability to differentiate into bone, cartilage, and stroma.^[^ ^8^, ^9^ ^]^ It remains unclear whether SSCs can be utilized to treat IVDD. SSCs derived from the vertebral endplate^[^ ^10^ ^]^ and growth plate,^[^ ^11^ ^]^ which exhibit cartilaginous structures, may offer advantages over other stem cells for IVDD treatment.^[^ ^12^, ^13^ ^]^ However, the biggest challenge is the inability to obtain autologous vertebral SSCs. On the other hand, costal cartilage can be used for autologous cell transplantation,^[^ ^14^ ^]^ and the isolation of costal cartilage SSCs may offer a key strategy for treating IVDD.

Healthy intervertebral discs (IVDs) maintain hypoxic homeostasis, where the oxygen partial pressure is lower than that in other structures.^[^ ^15^, ^16^, ^17^ ^]^ After transplantation into the IVD, stem cells inevitably undergo an environmental transition from normoxia to hypoxia, which leads to cellular oxidative stress,^[^ ^18^, ^19^ ^]^ along with rapid accumulation of intracellular reactive oxygen species (ROS). The imbalance between the production and degradation of intracellular ROS leads to ferroptosis, an ROS‐dependent form of regulated cell death.^[^ ^20^ ^]^ Therefore, hypoxia not only places stem cells at risk of rapid senescence and near death^[^ ^18^, ^21^, ^22^ ^]^ but also severely weakens the efficacy of stem cell therapy for IVDD.^[^ ^23^ ^]^ To overcome this disadvantage, in vitro, hypoxia preconditioning has been used in stem cell therapy^[^ ^24^ ^]^ as an effective strategy for IVDD treatment,^[^ ^12^ ^]^ which may be related to ROS production and ferroptosis regulation. Progressive hypoxia (PH), a type of hypoxic preconditioning designed based on hypoxia preconditioning, may have the same function of regulating cell differentiation and ferroptosis as hypoxic preconditioning. However, its role and the specific mechanism in regulating stem cell differentiation have not yet been elucidated.

The cyclic guanosine monophosphate–adenosine monophosphate synthase (cGAS)/stimulator of interferon genes (STING) pathway has emerged as a critical mechanism for coupling DNA sensing to the induction of powerful innate immune defense programs.^[^ ^25^ ^]^ This pathway can promote the antitumor effect of dendritic cells^[^ ^26^ ^]^ and regulate neural stem/progenitor cell differentiation.^[^ ^27^ ^]^ In IVDD, it promotes DNA damage‐associated senescence,^[^ ^28^ ^]^ activates NLRP3 inflammasome‐mediated pyroptosis,^[^ ^29^ ^]^ increases ROS levels,^[^ ^30^ ^]^ promotes extracellular matrix degradation,^[^ ^31^ ^]^ aggravates nucleus pulposus cell (NPC) apoptosis,^[^ ^32^ ^]^ and increases cartilage endplate chondrocyte degeneration via iron overload.^[^ ^33^ ^]^ However, the role of cGAS/STING signaling in treating IVDD using SSCs remains unknown. It is also unclear whether targeting cGAS/STING modulates SSC ferroptosis and differentiation.

Hydrogel sustained‐release carriers are widely used in inhibiting IVDD.^[^ ^34^ ^]^ When a hydrogel sustained‐release nanoparticle carrier is functionalized with ROS or pH responsiveness, it can enhance drug release in the disease environment and improve the therapeutic effect.^[^ ^35^ ^]^ Due to the accumulation of ROS in IVDD and further increase of ROS in exogenous cells after entering IVDD,^[^ ^31^, ^34^ ^]^ ROS‐responsive hydrogel sustained‐release carriers are particularly beneficial for IVDD treatment. One such carrier can be prepared using hyaluronic acid methacrylate(HAMA), 3‐aminophenylboronic acid (3‐APBA) and polydopamine nanoparticles (PDA‐NPs) coated with therapeutic drug molecules.^[^ ^36^ ^]^ After cross‐linking PDA‐NPs with HAMA via the bridge of 3‐APBA, a ROS‐responsive borate ester bond is formed,^[^ ^37^ ^]^ and an ROS‐responsive hydrogel sustained‐release carrier can be prepared. After exogenous stem cells enter the hypoxic environment in the IVD, ROS production and programmed cell death increase. Therefore, the 3‐D sustained‐release culture carrier formed by cultivating SSCs in ROS‐responsive hydrogel sustained release carriers may be more conducive to alleviating SSCs damage and promoting SSCs’ treatment of IVDD.

In this study, we sought to improve the utilization efficiency of costal cartilage‐derived SSCs for the treatment of IVDD. Hypoxia causes excessive ROS accumulation via the cGAS/STING pathway and promotes hypoxia‐inducible factor‐1 alpha (HIF‐1α) expression, thus further aggravating ferroptosis caused by mitophagy. Progressive hypoxia (PH) preconditioning moderately increases ROS levels and promotes the differentiation of SSCs into nucleus pulposus‐like cells (NPLCs) via Wnt signaling pathway. Based on these findings, we designed a ROS‐responsive 3‐D sustained‐release HAMA hydrogel carrier (HAMA‐C‐176@PDA‐NPs/SSCs) via cross‐linking with PDA‐NPs coated with the STING inhibitor C‐176 and incorporating SSCs. This approach reduced ROS production and ferroptosis in SSCs by targeting the STING pathway, promoted the differentiation of SSCs into NPLCs, and improved the utilization efficiency of SSCs in the treatment of IVDD.

## 2 Results

### 2.1 PH‐Preconditioned SSCs Effectively Delay IVDD

**Figure 1:**
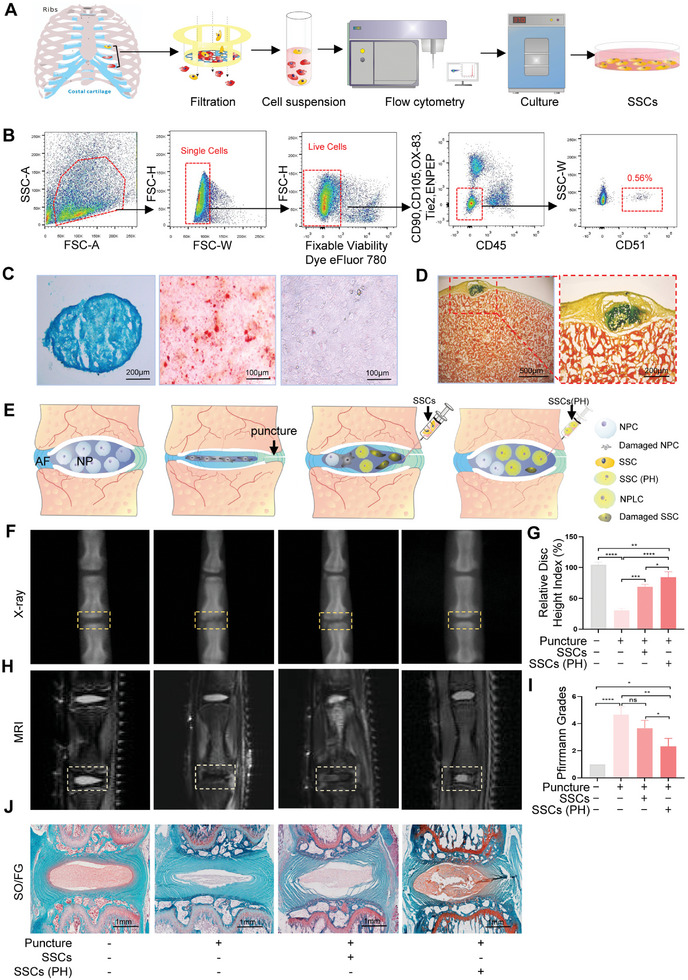
SSCs pretreated with PH effectively delay IVDD. A) Flow chart of SSC extraction from the costal cartilage of rats. Costal cartilage was obtained from 1–2‐week‐old rats and cut into 1–2 mm^3^ samples. A mixture of 2 mg mL^−1^ collagenase 2 and collagenase 4 was added for digestion for 1–2 h and then filtered through a 40 µm filter. Finally, the SSCs from the filtered cell suspension were sorted by flow cytometry and cultured in the incubator. B) Sorting and identification of the costal cartilage‐derived stem cells by flow cytometry, labeled CD90^−^CD45^−^CD105^−^ENPEP^−^Tie2^−^OX‐83^−^CD51^+^. C) Identification of chondrogenic and osteogenic potential and lack of adipogenic potential of SSCs using Alcian blue (left), alizarin red (middle), and Oil Red O (right) staining. D) Sectioned grafts stained with Movat's pentachrome. E) Treatment of IVDD after SSCs were cultured under normoxia or PH preconditioning. F, G) X‐ray and statistical results of the height of IVDs in the NC group (*n* = 3), puncture group (*n* = 3), puncture + SSCs group (*n* = 3), or puncture + PH‐preconditioned SSCs group (*n* = 3). H, I) MRI and statistical results of Pfirrmann grades. J) Safranin O‐fast green staining in NC, puncture, puncture + SSCs, or puncture + PH‐preconditioned SSCs IVDs. PH: progressive hypoxia. NP: nucleus pulposus; AF: annulus fibrosus; SSCs: skeletal stem cells; NPLC: nuclear pulposus‐like cell. Data in G, I) are presented as the mean ± SD, *p < 0.05, **p < 0.01, ***p < 0.001, ****p < 0.0001; ns, no significant difference by one‐way analysis of variance (ANOVA) test.

Whether SSCs are present in the costal cartilage is unknown. In this study, we extracted SSCs from rat costal cartilage as shown in **Figure** **1**A. According to the surface markers of SSCs,^[^ ^8^, ^9^, ^11^ ^]^ such as CD90^−^CD45^−^CD105^−^6C3^−^Tie2^−^Ter119^−^CD51^+^, we obtained a subpopulation of SSCs labeled as CD90^−^CD45^−^CD105^−^ENPEP^−^Tie2^−^OX‐83^−^CD51^+^ (Figure 1B). After inducing differentiation in vitro, the SSCs exhibited the potential to differentiate into chondrocytes and osteocytes but not adipocytes (Figure 1C). The SSCs were then transferred to the subcapsular region of the kidney, and differentiation into chondroid cells was observed after 2–4 weeks (Figure 1D). PH was performed, and its effect on SSCs was compared with that of hypoxia. The survival rate of SSCs in PH group was higher than that in hypoxia group (Figure S1A, Supporting Information). The Western blotting results showed that the expression levels of COL2, GLUT1, KRT19, and CA3 in the PH group were higher than those in the hypoxia group. Thus, PH was more favorable than hypoxia for SSCs to adapt to a hypoxic environment and to differentiate into NPLCs (Figure S1B, Supporting Information). Considering this finding and the hypoxic microenvironment of the IVD, SSCs were cultured under normoxia or PH preconditioning and then transplanted into the IVD. The difference in their effects on attenuating IVDD was analyzed (Figure 1E). X‐ray and statistical analyses indicated that SSCs cultured under PH preconditioning were more effective in increasing the height of the IVDs in puncture‐induced IVDD compared to those cultured under normoxia (Figure 1F,G). Magnetic resonance imaging (MRI) and statistical analysis of Pfirrmann grades also suggested that SSCs cultured with PH preconditioning effectively delayed IVDD (Figure 1H, I). Subsequently, IVD tissues were stained with safranin O‐fast green. After the puncture, the nucleus pulposus area in the IVD decreased, however, this area significantly increased after treatment with SSCs, especially PH‐preconditioned SSCs (Figure 1J). Thus, PH‐preconditioned SSCs exhibited a better therapeutic effect on IVDD.

### 2.2 PH Preconditioning Improves SSC Survival by Attenuating Ferroptosis

**Figure 2:**
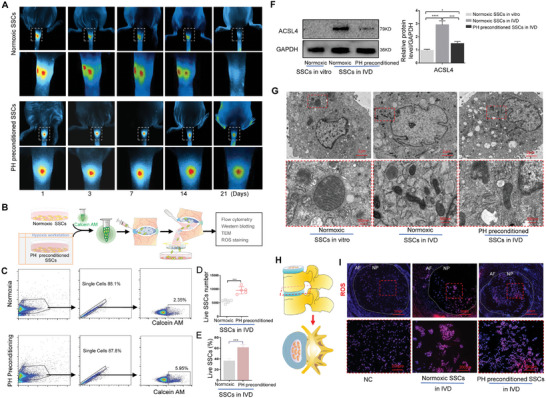
PH preconditioning improves the viability of SSCs after injection into IVDs. A) After culturing under normoxia or PH preconditioning, DIR‐labeled SSCs were injected into the IVDs of rats and imaged in vivo at different time points (*n* = 6). The six rats were divided into two groups of three. B) Flowchart of SSC counting and ferroptosis detection in the IVDs. Ten rats were divided into two groups of five. SSCs were cultured under normoxia or PH preconditioning and then stained with Calcein‐AM and injected into the IVDs. Five or six IVDs were selected from the tail of each rat for SSC injection. After 2–3 day, the rats were euthanized, the IVDs were opened, and the tissues and cells in the nucleus pulposus region were gently dissociated with PBS and collected in PBS solution. After gentle shaking and filtration through a 40 µm filter, a single SSC suspension was obtained. Cell precipitate containing a large number of SSCs was obtained by centrifugation, and then flow cytometry, western blotting, and transmission electron microscopy (TEM) were performed. C) Flow cytometry results for SSC survival after entering the IVD. ≈15 000 Calcein‐AM positive SSCs were injected into the IVDs. After 2–3 day, all cells in the IVDs were obtained and filtered through a 40 µm filter. Cell pellets was obtained by centrifugation and resuspended in 500 µL PBS. Flow cytometry was used to collect 50000 cells and calculate the volume *V* of the remaining cell suspension. The number *N* of Calcein‐AM‐positive SSCs was calculated based on the positive ratio *R*. Thus, the number of remained Calcein‐AM‐positive SSCs in the IVD after 2–3 days was calculated as *N* = 50 000 × *R*×500/(500–*V*). D, E) Statistical analysis of the number and proportion of remaining Calcein‐AM‐positive SSCs in the IVDs. F) Western blotting and quantitative protein levels of ACSL4 in SSCs. The “Normoxic SSCs in vitro” group was derived from the SSCs cultured under normoxic conditions in vitro. The “Normoxic SSCs in IVD” group was derived from SSCs injected into the intervertebral disc after normoxic culture in vitro. The “PH preconditioned SSCs in IVD” group was derived from SSCs injected into the intervertebral disc after PH preconditioning in vitro. G) TEM detection of mitochondrial damage in different SSC treatment groups. H) Diagram showing the preparation of tissue slices via IVD transection. I) Detection of ROS in transected IVD tissue slices after injection with phosphate‐buffered saline (NC), normoxic SSCs, or PH‐conditioned SSCs. NC: Normal Control. Data in D‐E) are presented as the mean ± SD, ***p < 0.001 by two‐tailed Student’s t‐test.  Data in F) are presented as the mean ± SD, *p < 0.05; **p < 0.01, ***p < 0.001, ****p < 0.0001 by one‐way ANOVA.

To explore the reasons for the effect of SSCs on IVDD, we analyzed changes in the number of SSCs after they entered the IVD. After being labeled with 1,1′‐dioctadecyl‐3,3,3′,3′‐tetramethylindotricarbocyanine iodide (DIR) and injected into the IVD, PH‐preconditioned SSCs showed a stronger fluorescence signal and intensity over time compared to normoxic SSCs, especially at 21 days (**Figure** **2**A). Subsequently, Calcein‐AM (acetoxymethyl)‐labeled SSCs were injected into the IVD, and follow‐up experiments were performed on days 2–3, as shown in Figure 2B. Flow cytometry analysis showed that the number of PH‐preconditioned SSCs was higher and their survival time longer in the IVD group than in the normoxia group. After injecting 15 000 SSCs into the IVD, approximately two‐thirds of the PH‐preconditioned SSCs and one‐third of the normoxic SSCs survived (Figure 2C–E). The western blotting results suggested that compared with SSCs cultured under normoxia in vitro, ferroptosis was enhanced in both normoxic and PH‐preconditioned SSCs after entering the IVD, especially in the normoxic SSCs (Figure 2F). The mitochondria of SSCs cultured under in vitro normoxia were visibly shrunken and deeply stained after injection into the IVD (Figure 2G). We also examined ROS production in the IVD after the injection of phosphate‐buffered saline (PBS) (NC), normoxic SSCs, or PH‐preconditioned SSCs. IVDs were transected, and frozen tissue slices were prepared (Figure 2H). Fluorescence intensity indicated that ROS production increased in the IVDs injected with normoxic SSCs or PH‐preconditioned SSCs, more so in the former (Figure 2I). Subsequently, we detected STING expression in IVD tissues, and the qPCR results suggested that compared with that in SSCs cultured under normoxia in vitro, the mRNA expression of *sting* expression increased in both normoxic and PH‐preconditioned SSCs after entering the IVD (Figure S2A, Supporting Information), which suggested that ferroptosis in SSCs entering the IVD might be closely related to STING.

**Figure 3:**
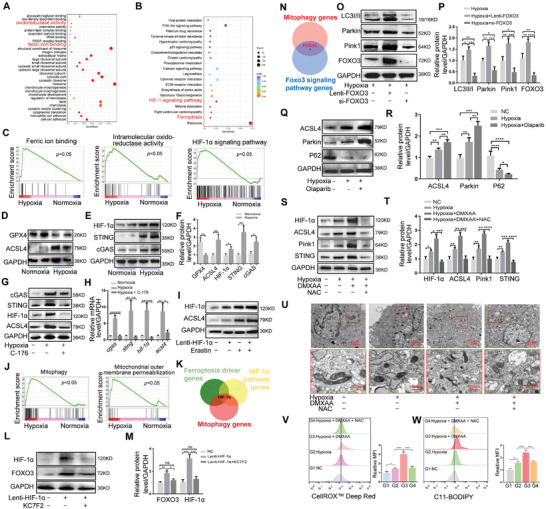
cGAS/STING aggravates ferroptosis in SSCs via the HIF‐1α/mitophagy axis. A–C) GO, KEGG, and GSEA analyses of genes in SSCs under hypoxia and normoxia. D–F) Western blotting and quantified protein levels of ACSL4, GPX4, HIF‐1α, STING, and cGAS in SSCs treated with hypoxia or normoxia. G, H) Representative Western blotting and mRNA levels of ACSL4, STING, cGAS, and HIF‐1α in SSCs in the NC, hypoxia, and hypoxia + C‐176 (5 µm) groups. I) Representative Western blotting of ACSL4 and HIF‐1α in SSCs in the NC, hypoxia, erastin, and hypoxia + erastin (10 µm) groups. J) GSEA analysis of SSCs cultured under normoxia and hypoxia. K) Venn diagram of ferroptosis driver genes, mitophagy genes, and HlF‐1α pathway genes. L, M) Western blotting and quantitative protein levels of FOXO3 and HIF‐1α in SSCs in the NC, Lenti‐HIF‐1α, and Lenti‐HIF‐1α + KC7F2 groups. N) Venn diagram of mitophagy genes and FOXO3 signaling pathway genes. O, P) Western blotting and quantitative protein levels of LC3II/I, PARKIN, PINK1, and FOXO3 in SSCs treated with hypoxia, hypoxia + Lenti‐FOXO3, or hypoxia + si‐FOXO3. Q, R) Western blotting and quantitative protein levels of ACSL4, PARKIN, and P62 in SSCs in the NC, hypoxia, and hypoxia + Olaparib groups. S, T) Western blotting and quantitative protein levels of HIF‐1α, ACSL4, Pink1, and STING in SSCs in the NC, hypoxia, hypoxia + DMXAA, and hypoxia + DMXAA + NAC groups. U) Detection of mitochondrial damage in different SSC treatment groups using TEM. V) CellROX Deep Red staining for lipid peroxidation in SSCs in the NC, hypoxia, hypoxia + DMXAA, and hypoxia + DMXAA + NAC groups. W) C11‐BODIPY staining for ROS levels in different SSC treatment groups. Data in F) are presented as the mean ± SD, *p < 0.05, **p < 0.01, ***p < 0.001 by two‐tailed Student's *t*‐test. Data in H, M, P, R, T, V, W) are presented as the mean ± SD, *p < 0.05, **p < 0.01, ***p < 0.001; ns, no significant difference by one‐way ANOVA.

### 2.3 cGAS/STING Activates HIF‐1α/FOXO3 via ROS Accumulation and Promotes Mitophagy‐Induced Ferroptosis

To simulate the hypoxic environment of the IVD, we cultured SSCs in 1% oxygen for 2–3 days, followed by RNA sequencing (RNA‐seq). Volcano plots revealed significant differences in gene expression between the hypoxic and normoxic groups (Figure S3A, Supporting Information). The expression of *slc7a11* decreased, while that of *acsl4*, *hmox1*, *cgas*, *sting*, and h*if‐1α* increased in the hypoxic group relative to values in the normoxic group (Figure S3B, Supporting Information). In Gene Ontology (GO) and Kyoto Encyclopedia of Genes and Genomes (KEGG) analyses, cell functions such as oxidoreductase activity and ferric iron binding were enriched, as were signaling pathways such as HlF‐1α and ferroptosis (**Figure** **3**A,B). Gene set enrichment analysis (GSEA) showed that ferric ion binding, intramolecular oxidoreductase activity, and HIF‐1α signaling pathway‐related genes were also significantly enriched in hypoxia‐cultured SSCs (Figure 3C). Therefore, the occurrence of ferroptosis in the hypoxia group may be related to the cGAS/STING and HIF‐1α pathways. Western blotting was used to verify differences in protein expression between the normoxic and hypoxic groups. Compared with the normoxic group, GPX4 expression was decreased and ACSL4, HIF‐1α, STING, and cGAS expression was increased in the hypoxic group (Figure 3D–F). The STING inhibitor C‐176 was then used to treat SSCs cultured under hypoxic conditions. Western blotting and quantitative reverse transcription polymerase chain reaction (RT‐qPCR) showed that hypoxia could increase the expression of HIF‐1α, cGAS, and STING, however, the expression of HIF‐1α was significantly decreased after the inhibition of the cGAS/STING signaling pathway, indicating that activation of this pathway promoted HIF‐1α expression (Figure 3G,H). HIF‐1α was overexpressed in SSCs using a lentivirus expressing HIF‐1α (Lenti‐HIF‐1α). Western blotting showed that Lenti‐HIF‐1α effectively promoted HIF‐1α expression (Figure S4, Supporting Information). To analyze the role of HIF‐1α in regulating ferroptosis, erastin (10 µm) was used to induce ferroptosis in SSCs. SSCs were subsequently treated with PBS (normal control [NC]), erastin, Lenti‐HIF‐1α, or erastin + Lenti‐HIF‐1α. Western blotting results showed that HIF‐1α not only increased ACSL4 expression but also enhanced erastin‐induced ACSL4 expression (Figure 3I). The oxidative stress detected via CellROX Deep Red staining in SSCs increased in Lenti‐HIF‐1α group, erastin group or erastin + Lenti‐HIF‐1α group, especially in SSCs treated with erastin + Lenti‐HIF‐1α, indicating that HIF‐1α promoted erastin‐induced oxidative stress (Figure S5A,B, Supporting Information). Cell viability and GSH levels associated with ferroptosis showed the opposite trend to that of oxidative stress (Figure S5C,D, Supporting Information), whereas MDA and iron ions levels showed the same trend as oxidative stress (Figure S5E,F, Supporting Information).

Hypoxia induces ferroptosis in a variety of cells,^[^ ^38^, ^39^ ^]^ but the role of HIF‐1α in regulating ferroptosis in SSCs in the IVD is not entirely clear. GSEA showed that mitophagy and mitochondrial outer membrane permeabilization‐related genes were enriched in the hypoxia group (Figure 3J). Therefore, hypoxia‐induced ferroptosis may be associated with mitophagy. Moreover, we found that ferroptosis driver genes, mitophagy genes, and HIF‐1α pathway genes shared a common gene, *Hif‐1α* (Figure 3K). Protein interactions between HIF‐1α, FOXO3, the mitophagy‐related proteins PARKIN/PINK1, and the ferroptosis‐related proteins GPX4/ACSL4/Hmox1/SLC7A11/Ftl1 were analyzed (Figure S6A, Supporting Information). The combined score of HIF‐1α and FOXO3 was 0.988 (Figure S6B, Supporting Information), which indicated that HIF‐1α may promote mitophagy by regulating FOXO3. Therefore, follow‐up experiments were conducted to test this hypothesis. Western blotting showed that the protein levels of HIF‐1α, FOXO3 increased with HIF‐1α overexpression via Lenti‐HIF‐1α and decreased with HIF‐1α inhibition via KC7F2 (Figure 3L,M). Subsequently, mitophagy and FOXO3 signaling pathway genes were analyzed, and 18 common genes, including *Foxo3*, were found to be shared between the mitophagy and FOXO3 signaling pathway genes, suggesting that FOXO3 plays a crucial role in regulating mitophagy (Figure 3N). The protein levels of FOXO3 in SSCs were increased or decreased by treatment with Lenti‐FOXO3 or small interfering (si)‐FOXO3, respectively (Figure S7, Supporting Information). To analyze the role of FOXO3 in the regulation of mitophagy, SSCs were subjected to hypoxia, hypoxia + Lenti‐FOXO3, or hypoxia + si‐FOXO3. Western blotting and statistical analysis suggested that FOXO3 promotes mitophagy (Figure 3O,P). After treating SSCs with hypoxia or hypoxia + olaparib, Western blotting results showed that olaparib, an agonist of mitophagy, increased the expression of PARKIN and ACSL4 induced by hypoxia and decreased the expression of P62 (Figure 3Q,R). ROS staining showed that the hypoxia‐induced increase in ROS production was exacerbated by olaparib (Figure S8A,B, Supporting Information). Cell viability and GSH levels decreased, while MDA and intracellular iron ions levels increased in the hypoxia + olaparib group compared to those in the hypoxia group (Figure S8C–F, Supporting Information).

Ferroptosis caused by hypoxia is closely related to an increase in intracellular oxidative stress and ROS levels.^[^ ^40^ ^]^ Considering the above results showing that hypoxia led to cGAS/STING activation, promoting ferroptosis, we further analyzed and verified whether ferroptosis induced by the cGAS/STING pathway was caused by an increase in ROS production as a result of oxidative stress. We treated SSCs with hypoxia, hypoxia + 5,6‐dimethylxanthenone‐4‐acetic acid (DMXAA), or hypoxia + DMXAA + NAC. Western blotting results showed that the expression of ACSL4, Pink1, HIF‐1α, and STING increased after SSCs were treated with the STING agonist DMXAA but decreased after treatment with the ROS inhibitor N‐acetylcysteine (NAC) compared with those in SSCs treated with hypoxia alone (Figure 3S,T). Compared with those in the hypoxia group, cell viability and GSH levels were decreased in the hypoxia + DMXAA group but increased in the hypoxia +DMXAA + NAC group, and MDA levels were increased in the hypoxia + DMXAA group but decreased in the hypoxia +DMXAA + NAC group (Figure S9, Supporting Information). TEM images also indicated that mitochondria under hypoxia were smaller and denser, which could be alleviated by NAC but was aggravated by DMXAA, compared with NC (Figure 3U). CellROX Deep Red Reagent staining indicated that DMXAA increased hypoxia‐induced oxidative stress, whereas NAC inhibited it (Figure 3V). C11‐BODIPY staining showed changes in intracellular ROS levels consistent with the observed trends in oxidative stress (Figure 3W).

### 2.4 Targeting STING Attenuates ROS Accumulation Under Hypoxia and Activates Wnt, Promoting SSC‐to‐NPLC Differentiation

**Figure 4:**
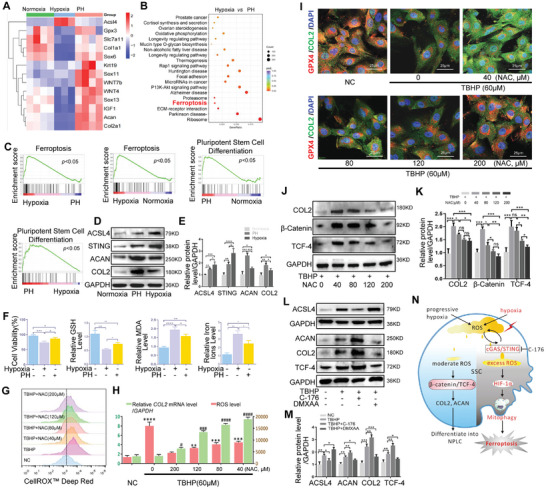
PH reduces ROS levels and promotes the differentiation of SSCs into NPLCs by downregulating STING expression. A) Heatmap analysis of SSCs cultured under normoxia, hypoxia, or PH preconditioning. B) KEGG analysis of SSCs cultured under hypoxia or PH preconditioning. C) GSEA analysis of SSCs cultured under NC, hypoxia, or PH preconditioning. D, E) Western blotting and quantitative protein levels of ACSL4, STING, ACAN, and COL2 in SSCs treated with NC, hypoxia, or PH preconditioning. F) Cell viability and levels of GSH, MDA, and intracellular iron ions detected in SSCs treated with NC, hypoxia, or PH preconditioning. G) CellROX Deep Red staining in SSCs treated with TBHP and different concentrations of NAC (0, 40, 80, 120, or 200 µm). H) Mean fluorescence intensity (MFI) of ROS and mRNA levels and statistical analysis in SSCs treated with TBHP and different concentrations of NAC (0, 40, 80, 120, or 200 µm) compared with NC. I) Immunofluorescence staining of COL2/GPX4 in SSCs treated with TBHP and different concentrations of NAC (0, 40, 80, 120, or 200 µm). J, K) Western blotting and quantitative protein levels of TCF‐4, β‐Catenin, and COL2 in SSCs treated with TBHP (60 µm) and NAC (0, 40, 80, 120, or 200 µm). L, M) Western blotting and quantitative protein levels of ACSL4, TCF‐4, COL2, and ACAN in SSCs in the NC, TBHP, TBHP + C‐176, and TBHP + DMXAA groups. N) Schematic of the cGAS/STING regulation of ferroptosis and differentiation under hypoxia or PH. Data in E, F, H, K, M) are presented as the mean ± SD, *p and ^#^ *p <* 0.05, **p and ^##^ *p <* 0.01, ***p and ^###^ *p <* 0.001, ****p and ^####^ *p <* 0.0001, ns, no significant difference by one‐way ANOVA.

According to the earlier results, PH‐preconditioned SSCs were more effective in treating IVDD than SSCs directly transferred to the IVD, but the underlying mechanism was not clear. SSCs were treated with PH preconditioning or hypoxia in vitro to simulate the IVD microenvironment for IVDD treatment, and RNA‐seq analysis was performed. Heat map analysis showed that compared with the PH group, the expression of a*csl4* increased while that of *slc7a11* and *gpx3* decreased in SSCs treated with hypoxia (**Figure** **4**A). Genes related to the differentiation of NPLCs, such as *sox6*, *krt19*, *wnt7b*, *acan*, and *col2*, were most highly expressed in SSCs treated with PH preconditioning (Figure 4A). KEGG analysis showed that the ferroptosis signaling pathway was more activated in the hypoxic group than in the PH group (Figure 4B). GSEA showed that ferroptosis‐related genes were more enriched in the hypoxic group than in the NC and PH groups, while pluripotent stem cell differentiation‐related genes were significantly enriched in the PH group (Figure 4C). Western blotting and statistical analysis showed that ACSL4 and STING levels were increased in both the hypoxia and PH preconditioning groups compared with those in the normoxia group. The expression of the differentiation‐related proteins ACAN and COL2 was also increased in both the hypoxic and PH groups compared to control values, especially in the PH group (Figure 4D,E). Cell viability and GSH levels decreased after SSCs were treated with hypoxia or PH, especially in the hypoxia group. This trend was the opposite of that observed for MDA and intracellular iron ions levels (Figure 4F). CellROX Deep Red and fluorescent staining for ROS indicated that oxidative stress (Figure S10A, Supporting Information) and ROS production (Figure S10B,C, Supporting Information) increased markedly in the hypoxic group compared with those in the PH group. To confirm the relation between ROS and differentiation, we analyzed the expression of COL2 and GPX4 using tert‐butyl hydroperoxide (TBHP) to induce ROS production and NAC to reduce TBHP‐induced ROS production. The oxidative stress and ROS levels, mRNA expression of *Col2*, and immunofluorescence staining for COL2 and GPX4 showed that 200 µm NAC, an inhibitor of ROS, effectively reduced ROS levels and increased GPX4 expression in SSCs treated with TBHP (60 µm). When the concentration of NAC was decreased from 200 to 40 µm, GPX4 expression decreased, but ROS and COL2 levels continued to increase (Figure 4G–I). Moreover, immunofluorescence staining and qPCR analysis of related markers of NPCs, such as carbonic anhydrase 3 (CA3), glucose transporter‐1 (GLUT1), aggrecan (ACAN), collagen II (COL2), and keratin 19 (KRT19), showed that the increase in ROS induced by TBHP (20 or 40 µm) promoted the effective differentiation of SSCs into NPLCs. However, excessive increase in ROS induced by TBHP (60 µm) weakened the differentiation of SSCs into NPLCs (Figure S11A,B, Supporting Information). These findings suggest that SSCs in the PH group more effectively inhibited IVDD compared to those in the hypoxic group through the reduction of ferroptosis and the promotion of differentiation after reducting ROS concentration.

Compared with normoxia, PH preconditioning better promoted the differentiation of SSCs into NPLCs. The RNA‐seq results of the normoxic and PH groups were analyzed, and the volcano plot showed significant differences in gene expression between the two groups (Figure S12A, Supporting Information). In particular, the expression of Wnt signaling proteins such as WNT4, WNT5, WNT7, and TCF3 was higher in the PH group than in the normoxic group (Figure S12B, Supporting Information). Moreover, the GO analysis showed that the oxidoreductase activity and functions related to the collagen‐containing extracellular matrix, collagen trimer, and extracellular matrix synthesis were significantly enriched in the PH group (Figure S12C, Supporting Information). Therefore, the differentiation of PH‐treated SSCs into NPLCs may be achieved through the activation of the Wnt signaling pathway by ROS. After inducing an increase in intracellular ROS production, we used different concentrations of NAC to reduce it. Western blotting results showed that with the gradual increase in NAC concentration, the levels of TCF‐4, β‐catenin, and COL2 first increased and then gradually decreased in SSCs treated with TBHP (Figure 4J,K). Moreover, the expression of TCF‐4, COL2, and ACAN increased after treatment with Wnt agonist 1 (Figure S13A,B, Supporting Information). The mRNA expression of t*cf‐4, col2*, and *acan* was also elevated in SSCs treated with the Wnt agonist 1 (Figure S13C, Supporting Information). Elevated ROS levels, such as those induced by PH or observed in SSCs treated with TBHP (60 µm) + NAC (40 µm), activated the Wnt signaling pathway and promoted the differentiation of SSCs into NPLCs. After ROS expression was elevated by TBHP, DMXAA further increased oxidative stress and ROS levels, while C‐176 decreased them. ROS levels were highest in the TBHP + DMXAA group but decreased in the TBHP + C‐176 group compared with those in the TBHP group (Figure S14A, Supporting Information). Western blotting indicated that ACSL4 expression was highest in the TBHP + DMXAA group, and TCF‐4, COL2, and ACAN expression was highest in the TBHP + C‐176 group (Figure 4L,M). Immunofluorescence staining showed similar trends (Figure S14B,C, Supporting Information). In summary, hypoxia promoted the activation of STING and the excess production of ROS, thus activating the HIF‐1α/mitophagy pathway and promoting ferroptosis in SSCs. Compared with the hypoxia group, STING expression and ROS production were lower in the PH group, which promoted the differentiation of SSCs into NPLCs through the Wnt pathway (Figure 4N).

### 2.5 Preparation and Characterization of C‐176@PDA‐NPs

**Figure 5:**
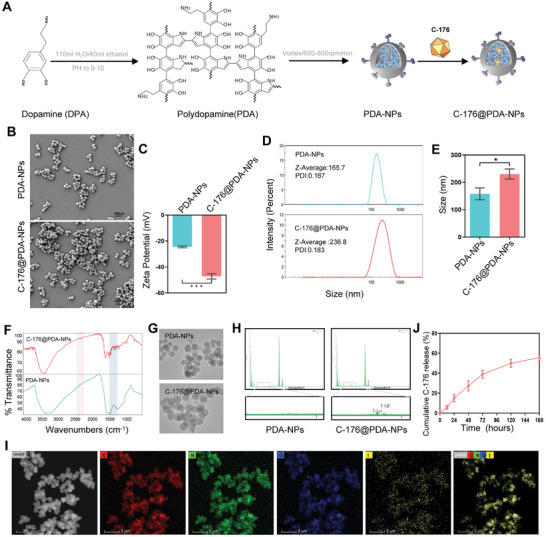
Preparation and characterization of C‐176@PDA‐NPs. A) Flowchart of the preparation of PDA‐NPs and C‐176@PDA‐NPs. B, C) Scanning electron microscopy and zeta potential analysis of PDA‐NPs and C‐176@PDA‐NPs. D) Particle size and polydispersity index (PDI) of PDA‐NPs and C‐176@PDA‐NPs detected using a zeta potential and particle size analyzer. E) Statistical analyses of particle sizes of PDA‐NPs and C‐176@PDA‐NPs. F) FTIR analysis of PDA‐NPs and C‐176@PDA‐NPs. G) TEM images of PDA‐NPs and C‐176@PDA‐NPs. H) Element analysis of PDA‐NPs and C‐176@PDA‐NPs via EDS. I) HAADF‐STEM and element mapping images of C‐176@PDA‐NPs. J) Cumulative release curve of C‐176 from C‐176@PDA‐NPs over time. Data in C, E) are presented as the mean ± SD; *p < 0.05, ***p < 0.001 by two‐tailed Student's *t*‐test.

PDA‐NPs coated with C‐176 (C‐176@PDA‐NPs) were designed and applied for the inhibition of IVDD. The preparation flow charts for PDA‐NPs and C‐176@PDA‐NPs are shown in **Figure** **5**A. PDA‐NPs and C‐176@PDA‐NPs were spheroidal (Figure 5B), and the Zeta Potential was obviously different (Figure 5C). The particle size of C‐176@PDA‐NPs was ≈236.8 nm and significantly larger than that of PDA‐NPs (Figure 5D,E). Moreover, Fourier transform infrared spectroscopy (FTIR) indicated several significant differences between PDA‐NPs and C‐176@PDA‐NPs at the 2300–2400 and 1200–1400 cm^−1^ bands (Figure 5F). Detection of the elements using TEM and energy dispersive spectrometry (EDS) showed that iodine was present in C‐176@PDA‐NPs but not in the PDA‐NPs (Figure 5G,H). By analyzing the high‐angle annular dark‐field scanning transmission electron microscopy (HAADF‐STEM) image and the corresponding EDS elemental mapping image, we found that iodine was encased inside the C‐176@PDA‐NPs (Figure 5I). The release of C‐176 gradually increased over time in C‐176@PDA‐NPs, and ≈55% of C‐176 was released within ≈7 days (Figure 5J). After preparating C‐176@PDA‐NPs, their role in regulating the ferroptosis and differentiation of SSCs was studied. Cell live/dead detection and statistical analysis indicated that PDA‐NPs and C‐176@PDA‐NPs were not cytotoxic to cells at 1 and 3 days (Figure S15A,B, Supporting Information). C‐176@PDA‐NPs significantly reduced the oxidative stress levels in SSCs, which had been elevated under hypoxia (Figure S15C, Supporting Information). ROS production showed the similar trend (Figure S15D,E, Supporting Information). Moreover, Western blotting and statistical analysis showed that C‐176@PDA‐NPs decreased the expression of STING and ACSL4 and increased the expression of COL2 and ACAN compared with that seen for PDA‐NPs (Figure S15F,G, Supporting Information). Thus, C‐176@PDA‐NPs reduced ROS levels in SSCs by inhibiting the STING pathway, attenuating ferroptosis, and promoting the differentiation of SSCs into NPLCs.

### 2.6 HAMA‐C‐176@PDA‐NPs Facilitate SSC‐to‐NPLC Differentiation by Releasing C‐176@PDA‐NPs and Targeting the cGAS/STING Pathway

**Figure 6:**
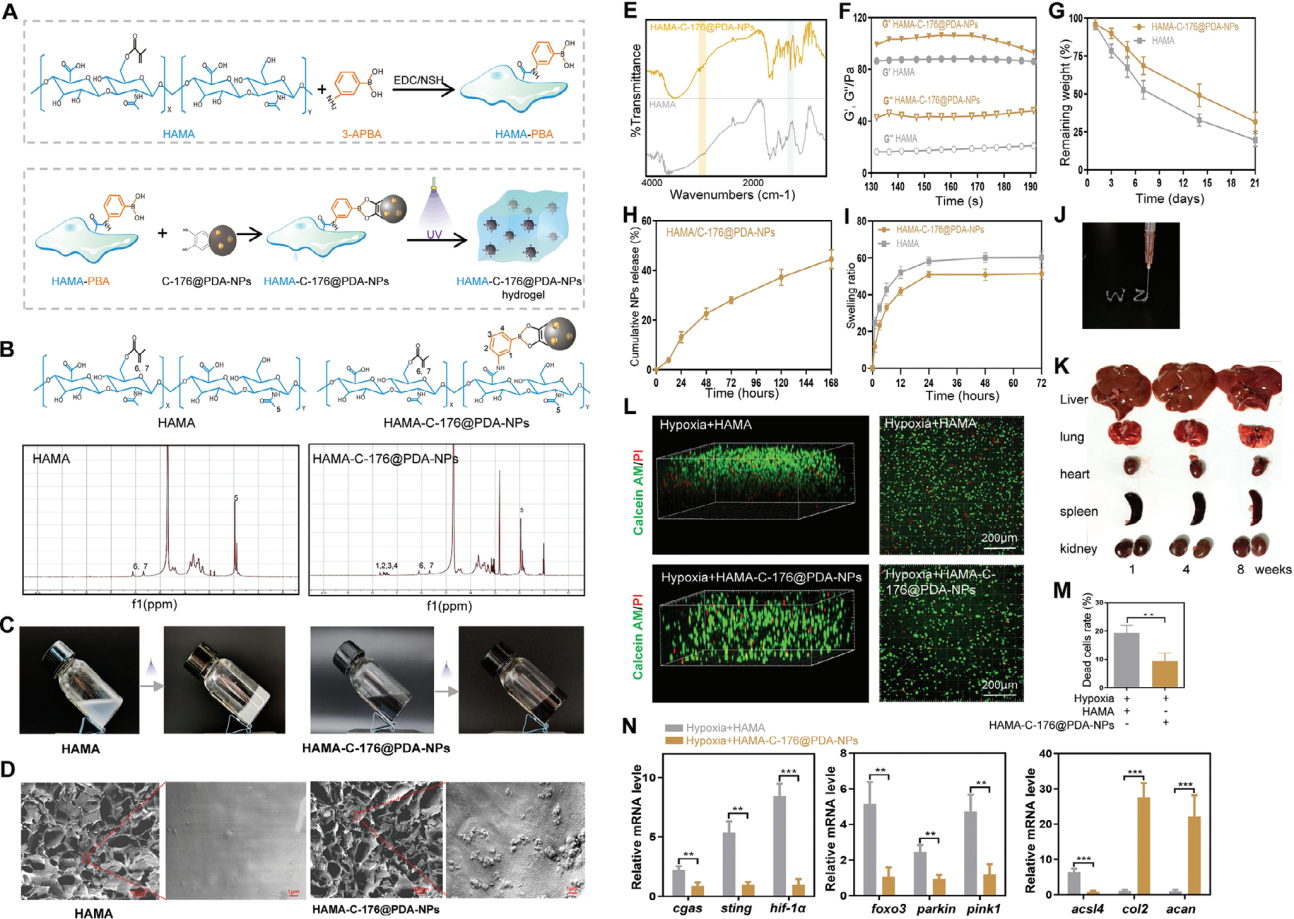
The 3‐D ROS‐responsive sustained‐release culture carrier targets the cGAS/STING pathway and promotes the differentiation of SSCs into NPLCs by releasing C‐176@PDA‐NPs. A) Flow chart of the preparation of HAMA and HAMA‐C‐176@PDA‐NPs. B) ^1^H‐NMR spectra of HAMA and HAMA‐C‐176@PDA‐NPs. C) HAMA and HAMA‐C‐176@PDA‐NPs hydrogels prepared by UV irradiation. D) Morphological and structural characteristics of the HAMA and HAMA‐C‐176@PDA‐NPs hydrogels and the distribution of PDA‐NPs as observed by SEM. E) FTIR spectra of the HAMA and HAMA‐C‐176@PDA‐NPs hydrogels. F) Rheological properties of HAMA and HAMA‐C‐176@PDA‐NPs hydrogels. G) Degradation curve of the HAMA and HAMA‐C‐176@PDA‐NPs hydrogels. H) Cumulative release curve of PDA‐NPs from the hydrogel. I) Swelling ratio of the HAMA and HAMA‐C‐176@PDA‐NPs hydrogels. J) Injectable hydrogel. K) Toxicity of the hydrogels at 1, 4, and 8 weeks. L, M) Live/dead staining and analysis of SSCs in the HAMA and HAMA‐C‐176@PDA‐NPs hydrogels. N) mRNA levels of *cgas, sting, hif‐1a, foxo3, parkin, pink1, acsl4, col2*, and a*can* in SSCs cultured in the hypoxia + HAMA hydrogel or hypoxia + HAMA‐C‐176@PDA‐NPs hydrogel. Data in M, N) are presented as the mean ± SD, *p < 0.05, **p < 0.01, ***p < 0.001, ****p < 0.0001 by two‐tailed Student's t‐test.

To regulate the balance of ferroptosis and differentiation in SSCs, we prepared a 3‐D ROS‐responsive sustained‐release culture carrier. 3‐Aminophenylboric acid (3‐APBA) acted as an intermediate bridge to cross‐link the HAMA hydrogel with C‐176@PDA‐NPs, thus forming a HAMA‐C‐176@PDA‐NPs hydrogel via ROS‐responsive borate ester bonds (**Figure** **6**A). The ^1^H NMR spectra of HAMA‐C‐176@PDA‐NPs indicated that HAMA and C‐176@PDA‐NPs were successfully cross‐linked (Figure 6B). Therefore, the HAMA‐C‐176@PDA‐NPs sustained‐release carrier could release C‐176@PDA‐NPs when the ROS concentration increased, target the STING pathway to reduce ROS levels, promote the differentiation of SSCs into NPLCs, and improve the inhibitory effect of SSCs on IVDD. The HAMA hydrogel and HAMA‐C‐176@PDA‐NPs hydrogel were in liquid form before UV irradiation and became hydrogel after 10–15 s of irradiation with UV light (Figure 6C). The surface structures of HAMA and HAMA‐C‐176@PDA‐NPs were analyzed by scanning electron microscopy. Although both were loose and porous, NPs were attached to the surface of the HAMA‐C‐176@PDA‐NPs hydrogel (Figure 6D). FTIR indicated several differences between HAMA and HAMA‐C‐176@PDA‐NPs ≈3000 and 1200 cm^−1^ bands (Figure 6E). The storage and loss moduli of HAMA and HAMA‐C‐176@PDA‐NPs are shown in Figure 6F. In the solution containing type I/II collagenase and hyaluronidase, the HAMA and HAMA‐C‐176@PDA‐NPs hydrogel gradually degraded, and the residual amount was ≈25–30% at 21 days (Figure 6G). After HAMA was mixed directly with PDA‐NPs without cross‐linking via the bridge of 3‐APBA, the release of PDA‐NPs was analyzed. Over time, PDA‐NPs were gradually released, with ≈45% released by day 7 (Figure 6H). The swelling ratios of HAMA and HAMA‐C‐176@NPs were ≈55–60 times at 72 h (Figure 6I). HAMA‐C‐176@PDA‐NPs were injectable and demonstrated non‐toxicity after observing the morphology of heart, liver, spleen, lung, and kidney in rats at 1, 4, and 8 weeks after injection (Figure 6J,K). To verify that the ROS‐responsive HAMA‐C‐176@PDA‐NPs hydrogel could release C‐176@PDA‐NPs under hypoxia to inhibit ferroptosis, HAMA or HAMA‐C‐176@PDA‐NPs hydrogel was mixed with SSCs (3‐D sustained‐release culture carrier) and then cultured under hypoxia. After 2–3 days of culture, the cells were stained with Calcein‐AM/propidium iodide reagent and imaged using a laser confocal microscope. Fluorescence observation and statistical results showed that the percentage of dead red blood cells was lower in the hypoxia + HAMA‐C‐176@PDA‐NPs group than in the hypoxia + HAMA group (Figure 6L,M). RT‐qPCR results showed that mRNA expression of *cgas*, *sting*, *hif1a*, *pink1*, *parkin*, and *foxo3* was higher in the hypoxia + HAMA group; however, that of *col2* and *acan* was higher in the hypoxia + HAMA‐C‐176@PDA‐NPs group (Figure 6N). It was further confirmed that the 3‐D ROS‐responsive sustained‐release culture carrier inhibited the expression of HIF‐1α and FOXO3 and the occurrence of mitophagy under hypoxia by inhibiting the cGAS/STING pathway, thereby inhibiting ferroptosis and promoting the differentiation of SSCs into NPLCs.

### 2.7 The 3‐D Sustained‐Release Culture Carrier Effectively Delays IVDD in Rats

**Figure 7:**
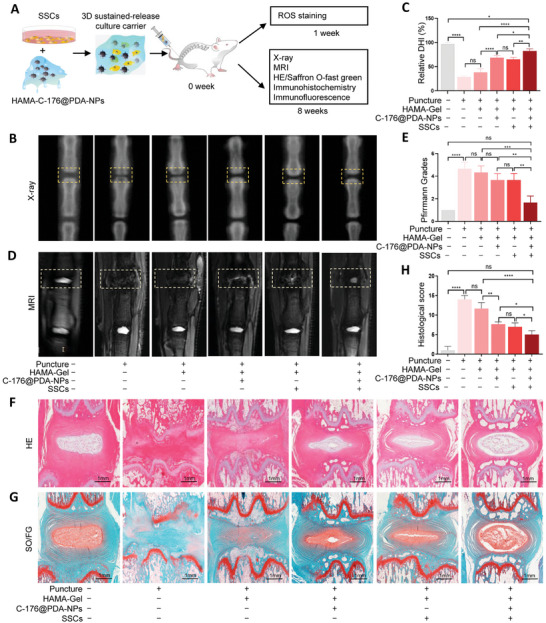
The 3‐D sustained‐release culture carrier effectively inhibits IVDD. A) Flow chart of the treatment of IVDD using the 3‐D sustained‐release culture carrier. The 3‐D sustained‐release culture carrier was constructed by mixing HAMA‐C‐176@PDA‐NPs hydrogel with SSCs. B, C) X‐ray images and statistical analysis of IVD height. D, E) Representative MRI results and Pfirrmann grades. F–H) HE staining, safranin O‐fast green staining, and histological scores of rat IVDs in the NC (*n* = 3), puncture (*n* = 3), puncture + HAMA hydrogel (*n* = 3), puncture + HAMA‐C‐176@PDA‐NPs (*n* = 3), puncture + HAMA hydrogel + SSCs (*n* = 3), and puncture + HAMA‐C‐176@PDA‐NPs hydrogel + SSCs (*n* = 3) groups. DHI: disc height index. Data in C, E, H) are presented as the mean ± SD *p < 0.05, **p < 0.01, ***p < 0.001, ****p < 0.0001, ns, no significant difference by one‐way ANOVA.

**Figure 8:**
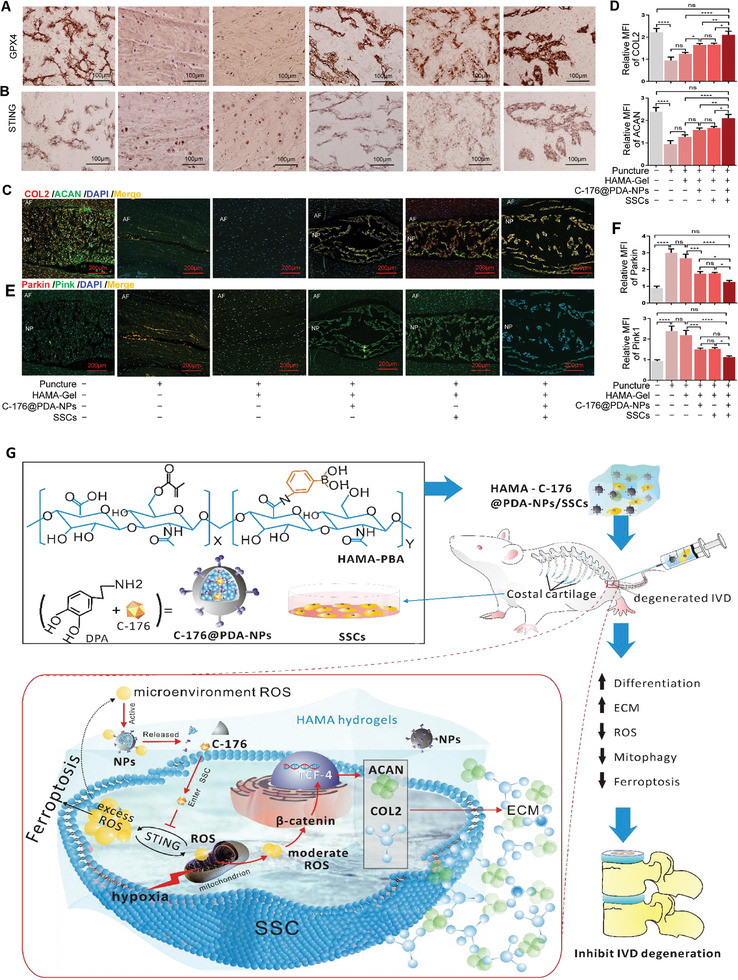
The 3‐D sustained‐release culture carrier reduces ROS production and mitophagy and promotes the differentiation of SSCs to attenuate IVDD by targeting STING. A, B) Immunohistochemical staining for STING and GPX4 in rat IVDs in the NC, puncture, puncture + HAMA hydrogel, puncture + HAMA‐C‐176@PDA‐NPs, puncture + HAMA hydrogel + SSCs, and puncture + HAMA‐C‐176@PDA‐NPs hydrogel + SSCs group. C, D) Immunofluorescence staining and quantitative analysis of the MFI of COL2 and ACAN in rat IVDs in the NC, puncture, puncture + HAMA hydrogel, puncture + HAMA‐C‐176@PDA‐NPs, puncture + HAMA hydrogel + SSCs, and puncture + HAMA‐C‐176@PDA‐NPs hydrogel + SSCs groups. E, F) Immunofluorescence staining and quantitative analysis of the MFI of Parkin and Pink1 in rat IVDs treated as above. G) Proposed mechanisms by which the 3‐D sustained‐release culture carrier prevents the accumulation of ROS by continuously releasing C‐176@PDA‐NPs and inhibiting cGAS–STING pathway activation due to sudden hypoxia, thereby preventing ferroptosis and promoting differentiation in costal cartilage‐derived SSCs, ultimately attenuating IVDD progression. Data in D, F) are presented as the mean ± SD, *p < 0.05, **p < 0.01, ***p < 0.001, ****p < 0.0001, ns, no significant difference by one‐way ANOVA.

To verify whether the 3‐D sustained‐release culture carrier constructed by mixing HAMA‐C‐176@PDA‐NPs and SSCs reduced ROS production and promoted the differentiation of SSCs in vivo, rats were divided into the following treatment groups: NC, puncture, puncture + HAMA hydrogel, puncture + HAMA‐C‐176@PDA‐NPs, puncture + HAMA hydrogel + SSCs, and puncture + 3‐D sustained‐release culture carrier (HAMA‐C‐176@PDA‐NPs hydrogel + SSCs) groups. In the first week, eighteen rats were stained for ROS. The remaining rats were then evaluated by X‐ray, micro‐MRI, and histological staining at 8 weeks after surgery and treatment (**Figure** **7**A). ROS levels were significantly increased in the puncture, puncture + HAMA, and puncture + HAMA + SSCs groups, but the increase was significantly inhibited by the intradiscal injection of HAMA‐C‐176@PDA‐NPs and HAMA‐C‐176@PDA‐NPs + SSCs (Figure S16A,B, Supporting Information). X‐ray and statistical analyses showed that IVD height decreased in all groups after puncture, but the HAMA‐C‐176@PDA‐NPs + SSCs group better maintained IVD height (Figure 7B,C). MRI and Pfirrmann grade results suggested that HAMA‐C‐176@PDA‐NPs + SSCs significantly ameliorated IVDD in rats (Figure 7D,E). As shown in Figure 7F–H, the results of hematoxylin and eosin and safranin O‐fast green staining and histological scores indicated that the height and area of the nucleus pulposus region were significantly reduced in the puncture and HAMA groups compared with those in the NC group. After treatment with intradiscal injection of HAMA‐C‐176@PDA‐NPs, HAMA + SSCs or HAMA‐C‐176@PDA‐NPs + SSCs, the height and area of the nucleus pulposus region increased, and the structure of the annulus was more complete, especially after the latter treatment. Histochemical staining showed that GPX4 expression increased significantly whereas STING expression decreased in the puncture + HAMA‐C‐176@PDA‐NPs + SSCs group compared with that in the puncture, puncture + HAMA, puncture + HAMA‐C‐176@PDA‐NPs and puncture + HAMA + SSCs groups (**Figure** **8**A,B). Histological immunofluorescence staining and statistical analysis of MFI also showed that COL2 and ACAN expression increased significantly (Figure 8E,F) but Parkin and Pink1 expression decreased in rats treated with puncture + HAMA‐C‐176@PDA‐NPs + SSCs (Figure 8C,D). In summary, the 3‐D sustained‐release culture carrier (HAMA‐C‐176@PDA‐NPs hydrogel + SSCs) ameliorated IVDD by reducing ROS levels, inhibiting ferroptosis, and promoting SSC differentiation, thereby attenuating IVDD in vivo (Figure 8G).

## 3 Discussion

Studies have shown that when stem cells are injected into degenerated IVDs using minimally invasive regenerative therapy,^[^ ^41^ ^]^ they supplement the lost NPCs and release extracellular matrix to combat degeneration. This phenomenon is widely studied and considered a promising clinical therapeutic strategy. However, the nucleus pulposus is a special structure in a hypoxic state,^[^ ^42^, ^43^ ^]^ which poses an extremely harsh microenvironment for transplanted stem cells. Therefore, many issues need to be addressed to improve the utilization of stem cells in the treatment of IVDD. Stem cells have been extensively used in preclinical research and in the clinical treatment of various diseases such as age‐related diseases,^[^ ^44^ ^]^ tumors,^[^ ^45^ ^]^ and tissue lesions.^[^ ^46^ ^]^ The oxygen partial pressure and nutrient supply of different tissues in the body vary significantly, posing significant challenges in terms of the survival of exogenous stem cell transplants. IVD homeostasis is maintained at an ≈1% oxygen concentration.^[^ ^47^ ^]^ Oxidative stress following hypoxia impairs the function of transplanted stem cells^[^ ^48^ ^]^ and increases ROS production and apoptosis.^[^ ^49^ ^]^ Therefore, it is imperative to elucidate the mechanism of stem cell repair and improve the efficiency of stem cell utilization in the hypoxic environment of IVDD.

SSCs are important in the healing and repair of fractures and joint injuries.^[^ ^9^, ^50^ ^]^ However, the role of SSCs in delaying IVDD has not been extensively studied. Stem cells derived from chondrocytes are more effective than mesenchymal stem cells (MSCs) in repairing IVDD.^[^ ^13^ ^]^ In this study, SSCs from the costal cartilage of rats were extracted and identified based on surface markers similar to those of mouse and human SSCs.^[^ ^8^, ^11^, ^51^ ^]^ Previous experimental data have shown that the hypoxic cultivation of MSCs can improve their efficacy in disease treatment. Therefore, we used SSCs preconditioned with PH to treat IVDD. The results showed that SSCs inhibited IVDD progression, and those preconditioned with PH were more effective for IVDD repair. Moreover, SSCs preconditioned with hypoxia survived longer in the IVD with higher survival rates. The reason for this may be that hypoxia increased apoptosis and ferroptosis in unprocessed SSCs more compared to PH‐preconditioned SSCs;^[^ ^52^ ^]^ however, we believe that this may only provide a partial explanation.

STING promotes ferroptosis in a variety of diseases,^[^ ^32^, ^53^, ^54^ ^]^ however, it remains unclear whether STING attenuates the inhibitory effect of SSCs on IVDD via ferroptosis regulation and its related mechanisms after SSCs enter the IVD. To explore these mechanisms, SSCs were cultured under normoxia or hypoxia in vitro to simulate the process of SSCs entering the IVD. The results showed that in the hypoxic group, cGAS/STING signaling activation increased the expression of HIF‐1α, leading to ferroptosis. Analysis of the shared genes and associated mechanisms revealed that HlF‐1α promoted the expression of FOXO3 to activate mitophagy. Mitophagy promotes SSC ferroptosis. Considering that cGAS/STING signaling pathway increases ROS production,^[^ ^30^, ^55^ ^]^ we analyzed whether the cGAS/STING signaling pathway also regulates hypoxia‐induced ferroptosis by regulating ROS. The results showed that increased STING expression elevated oxidative stress and ROS concentration, as well as the expression of HIF‐1α, Pink1, and ACSL4, resulting in the ferroptosis of SSCs. Although STING is closely related to ferroptosis,^[^ ^54^ ^]^ previous studies have not clarified whether the weakened curative effect of SSCs on IVDD in a hypoxic environment was due to STING/HIF‐1α/mitophagy‐induced ferroptosis. In the current study, we clarified that STING could promote the occurrence of ferroptosis by increasing ROS production and subsequently activating the HIF‐1α/mitophagy axis. Based on our results, the STING/ROS/ferroptosis cascade signaling may be the main reason for inefficient stem cell transplantation in IVDs. Previous studies has shown that mitophagy alleviates IVDD,^[^ ^56^ ^]^ which is inconsistent with our findings that mitophagy promoted ferroptosis and weakened the repair effect of SSCs on IVDD. In other studies, such as ischemia‐reperfusion injury, hypoxia, or radiation, excessive mitophagy increases the release of iron ions or fatty acids, exacerbating ferroptosis.^[^ ^57^, ^58^, ^59^ ^]^ Thus, the discrepancy between our results and other studies may be due to the varying degrees of mitophagy flux and stress response.

In this study, PH‐preconditioned SSCs were particularly effective in inhibiting IVDD; however, the underlying mechanism was unclear. Reducing ROS generation can attenuate ischemia–reperfusion‐mediated remote lung injury,^[^ ^60^ ^]^ promote MSC survival, and inhibit myocardial infarction.^[^ ^61^ ^]^ Therefore, we hypothesized that after PH preconditioning, SSCs may also enhance the inhibitory effect on IVDD by reducing excessive ROS accumulation. The results of RNA‐seq, western blotting, and immunofluorescence staining indicated that compared with those in the normoxic culture, ferroptosis and STING expression in SSCs were significantly increased in both hypoxic and PH groups, with a more pronounced increase in the hypoxic group. Additionally, differentiation‐related genes, such as c*ol2* and a*can*, were significantly upregulated in both hypoxic and PH groups, especially in the PH group. Moreover, the expression of COL2 and ACAN increased with the increase in STING expression in the PH and hypoxia groups compared with that in the NC group, however, their expression decreased with the increase in STING expression in the hypoxia group compared with that in the PH group. In summary, PH reduced the occurrence of ferroptosis by weakening the expression of STING compared with that seen in the hypoxia group, thereby strengthening the differentiation process. Studies have showed that STING can not only promote T‐lymphocyte proliferation and differentiation,^[^ ^62^ ^]^ but also inhibits osteoblast and osteoclast differentiation^[^ ^63^ ^]^ and the neurogenic differentiation potential of neural stem cells.^[^ ^27^ ^]^ Therefore, we hypothesize that the cGAS/STING is also related to SSC differentiation, and the specific mechanism for promoting or inhibiting stem cell differentiation requires further study.

Studies have shown that hypoxia increases the occurrence of apoptosis^[^ ^64^ ^]^ and ferroptosis^[^ ^65^, ^66^ ^]^ and leads to extracellular matrix production and cell differentiation.^[^ ^67^ ^]^ Our results suggest that PH and hypoxia better promote the activation of the STING pathway and the expression of COL2 and ACAN than normoxia. The overactivation of STING pathway, in turn, weakened stem cell differentiation under hypoxic conditions. Moreover, previous studies have shown that cGAS/STING signaling is closely related to differentiation or ferroptosis.^[^ ^27^, ^54^ ^]^ Therefore, STING may play an important role in explaining how hypoxia promotes both ferroptosis and differentiation. STING also enhances ROS production.^[^ ^30^ ^]^ ROS, as a product of intracellular oxidative stress, not only promotes cell senescence and ferroptosis^[^ ^68^ ^]^ but also plays an important role in the proliferation and differentiation of tumor and non‐tumor stem cells.^[^ ^69^ ^]^ For example, ROS promotes the osteogenic differentiation of diabetic tendon stem/progenitor cells.^[^ ^70^ ^]^ Thus, ROS might be the key factor balancing in the STING regulation of ferroptosis and stem cell differentiation. To confirm this, TBHP was used to induce ROS production, and different concentrations of NAC were used to curb ROS accumulation. When oxidative stress and ROS production were inhibited, ferroptosis decreased significantly, whereas differentiation increased significantly. In addition, STING activation promoted ROS production and exacerbated ferroptosis. Correspondingly, ROS levels decreased, and differentiation was significantly enhanced after inhibiting STING via C‐176. Previous studies have shown that SING promotes T‐lymphocyte differentiation. In this study, the STING agonist DMXAA was added after inducing ROS production with TBHP, resulting in a large amount of ROS were produced and accumulated, which promoted the ACSL4 expression, aggravated ferroptosis, and weakened but did not completely inhibit stem cell differentiation. Based on these findings, we concluded that the increase in ROS induced by STING is a key factor in regulating the balance between ferroptosis and SSC differentiation in PH and hypoxia. However, we need to further clarify whether the inconsistent role of STING in stem cell differentiation is related with ROS accumulation.

The pathway mechanisms involved in differentiation were identified by analyzing the RNA‐seq results. The heat map results showed that PH‐induced differentiation may be related to the Wnt pathway. Western blotting and RT‐qPCR results also suggested that the differentiation of SSCs into NPLCs was enhanced by Wnt/β‐catenin/TCF4 signaling after the accumulation of ROS was curbed by NAC. Therefore, given the results of previous experiments and our findings, we concluded that PH preconditioning could increase the effect of SSCs on IVDD repair by reducing STING‐induced ROS production and activating Wnt/β‐Catenin/TCF4 signaling, thereby attenuating ferroptosis and promoting the differentiation of SSCs into NPLCs. In conclusion, consistent with previous research, we found that STING increased ROS production and ferroptosis in stem cells transplanted into the IVD.^[^ ^31^ ^]^ Importantly, targeting STING may enhance the differentiation of SSCs into NPLCs after entering the IVD, providing a clear direction for future research aimed at developing drugs that reduce STING expression.

Drug‐loaded NPs are widely used to treat various diseases because of their beneficial effects such as ROS depletion and inhibition of ferroptosis.^[^ ^71^ ^]^ Considering the increase in ROS produced by senescent and degenerative NPCs in IVDD^[^ ^71^ ^]^ and the further increase in ROS production from exogenous stem cells^[^ ^23^ ^]^ after SSCs enter the IVD, ROS‐responsive hydrogel sustained‐release carriers might be more effective than NPs for the treatment of IVDD. Moreover, cGAS/STING pathway activation and an increase in STING expression can lead to increased ROS production.^[^ ^30^ ^]^ Therefore, we designed an ROS‐responsive HAMA‐C‐176@PDA‐NPs hydrogel sustained‐release carrier that released C‐176@PDA‐NPs in response to elevated ROS levels. C‐176@PDA‐NPs effectively inhibited STING expression, thereby inhibiting the ferroptosis of SSCs and promoting their differentiation into NPLCs. In addition, the 3‐D sustained‐release culture carrier constructed by mixing the HAMA‐C‐176@PDA‐NPs hydrogel with SSCs effectively inhibited the ferroptosis of SSCs, promoted the differentiation of SSCs in vitro, and improved the inhibitory effect of SSCs on IVDD by targeting the cGAS/STING pathway in vivo. Several studies have been conducted on hydrogel carriers to reduce ROS production and delay IVDD.^[^ ^72^, ^73^ ^]^ However, the 3‐D sustained‐release culture carrier prepared based on a mechanism whereby PH preconditioning promotes the differentiation of SSCs into NPLCs is a novel contribution of this study; moreover, our results elucidate the mechanisms and applications of stem cells for improved treatment of IVDD.

## 4 Conclusion

Our study demonstrated that the activation or inhibition of cGAS/STING signaling can regulate ROS levels and influence the balance between ferroptosis and differentiation of SSCs in hypoxic environments. The novel 3‐D ROS‐responsive sustained‐release culture carrier introduced in this study provides a practical approach for improving the efficiency of SSCs in the treatment of IVDD. Future research should focus on the mechanisms and applications of cGAS/STING modulation to regulate stem cell differentiation and enhance its therapeutic effects on hypoxia‐related diseases.

## 5 Experimental Section

### Reagents and Antibodies

Olaparib (cat. no. HY‐10162) and the ferroptosis inducer erastin (cat. no. HY‐15763) were obtained from MedChemExpress (Shanghai, China). HIF‐1α signaling pathway inhibitor (KC7F2, cat. no. S7946), Wnt agonist 1 (cat. no. S8178), STING activator DMXAA (cat. no. S1537), ROS inhibitor NAC (cat. no. S1623), were purchased from Sellect (Shanghai, China). Type II collagenase (cat. no. A004174‐0001) and type IV collagenase (cat. no. A004186‐0100) were purchased from Sangon Biotech (Shanghai, China). Anti‐glyceraldehyde 3‐phosphate dehydrogenase (GAPDH) (cat. no. 60004‐1‐Ig), anti‐aggrecan (ACAN) (cat. no. 13880‐1‐AP), anti‐collagen type 2 (COL2) (cat. no. 28459‐1‐AP), anti‐GATA4 (cat. no. 19530‐1‐AP), anti‐STING (cat. no. 19851‐1‐AP), anti‐Parkin (cat. no. 14060‐1‐AP), anti‐Pink1 (cat. no. 23274‐1‐AP), anti‐P62 (cat. no. 18420‐1‐AP), anti‐LC3I/II (cat. no. 14600‐1‐AP), anti‐LC3B (cat. no. 18725‐1‐AP), anti‐GPX4 (cat. no. 67763‐1‐Ig), anti‐Tie2 (cat. no. 19157‐1‐AP), anti‐ENPEP (CD249 or 6C3) (cat. no. 17655‐1‐AP), 488‐conjugated goat anti‐rabbit IgG (cat. no. SA00013‐2), and 594‐conjugated goat anti‐mouse IgG (cat. no. SA00013‐3) antibodies were obtained from Proteintech (Wuhan, China). Anti‐HIF‐1α antibody (cat. no. SAB2702132) was purchased from Sigma‐Aldrich (St. Louis, MO, USA). Anti‐cGAS antibody (cat. no. sc‐515777) was purchased from Santa Cruz Biotechnology (Dallas, TX, USA). Anti‐SRY‐box containing gene 9 (SOX9) antibody was obtained from ABclonal (cat. no. A19710, Wuhan, China). Anti‐ACSL4 (cat. no. ab155282), goat anti‐rabbit IgG (cat. no. ab150080), and goat anti‐mouse IgG (cat. no. ab150113) antibodies were purchased from Abcam (Cambridge, MA, USA). Anti‐FOXO3 antibody was obtained from Cell Signaling Technology (cat. no. 2497S; Danvers, MA, USA). Anti‐transcription factor 4 (TCF4) (cat. no. A00674‐2) and anti‐β‐catenin (cat. no. BA0426) antibodies were obtained from Boster (Wuhan, China). Anti‐CD90 (cat. no. 206 105), anti‐CD45 (cat. no. 202 220), and anti‐CD51 (ITGAV) (cat. no. 920 007) antibodies were purchased from BioLegend (San Diego, CA, USA). eBioscienc Fixable Viability Dye eFluo 780 (cat. no. 65‐0865‐14), anti‐CD105 (cat. no. MA1‐19594), and anti‐OX‐83 (erythroid cell marker, named OX‐83 in rats and Ter119 in mice) (cat. no. MA5‐17580) antibodies were purchased from Thermo Fisher Scientific (Waltham, MA, USA). Alcian blue (cat. no. G1562), alizarin red (cat. no. G8550), and oil red O (cat. no. G1260) were purchased from Solarbio (Beijing, China).

### SSC Isolation and Identification

SSCs were isolated from the costal cartilage of 1–2‐week‐old Sprague–Dawley rats as previously reported.^[^ ^9^, ^10^ ^]^ The isolated costal cartilage tissues were not removed of costal perichondrium and cut into 1–2 mm^3^ tissue blocks and digested with 0.2% type II collagenase for 1–2 h at 37 °C. After filtering through a 40 µm filter screen, the SSCs in the cell suspension were sorted by flow cytometry according to surface markers CD90^−^CD45^−^CD105^−^ENPEP^−^Tie2^−^OX‐83^−^CD51^+^, and then cultured in stem cell complete culture medium (cat. no. CM‐R131, Procell, Tianjin, China) at 5% CO_2_ and 37 °C, and identified after cell fusion reached 80–90%.

### Osteogenic Differentiation

SSCs were seeded in 0.1% gelatin‐coated six‐well plates and cultured in Dulbecco's modified Eagle's medium with nutrient mixture F‐12 (DMEM/F12) containing 10% fetal bovine serum and 1% penicillin–streptomycin at 5% CO_2_ and 37 °C. At 70% confluence, the complete medium was removed, and 2 mL of MSC osteogenic induction medium (cat. no. MUBMX‐90021, Cyagen, Guangzhou, China) was added. The solution was changed every 3 days. After 2–4 weeks of induction, the cells were stained with alizarin red and imaged under a microscope (Olympus, Tokyo, Japan).

### Chondrogenic Differentiation

SSCs were transferred into a centrifuge tube and centrifuged at 250 × *g* for 5–10 min. The cell pellet was resuspended in 1 mL chondrogenic differentiation medium (cat. no. MUCMX‐9004; Cyagen) and recentrifuged. The centrifuge tube was then placed in an incubator for culture. After 48–72 h, the centrifuge tube was flicked to release the cartilage ball, which was suspended in the medium. The medium was replaced with 0.5 mL chondroblast induction and differentiation complete medium every 2–3 days. After continuous induction for 15–21 days, the cartilage balls were fixed overnight with 4% paraformaldehyde and subsequently prepared into frozen slices. Following PBS washes, tissue sections were stained with Alcian blue for 1 h and imaged using a microscope.

### Adipogenic Differentiation

SSCs were seeded in six‐well plates and cultured in incubators after the addition of complete medium. After the cells reached 100% confluence, the complete medium was replaced with adipogenic induction differentiation medium A (cat. no. MUBMX‐90031, Cyagen). After 3 days of culture, adipogenic induction differentiation medium A was removed, and adipogenic induction differentiation medium B was added. After 24 h, medium B was replaced with medium A for culture. After 1–2 weeks, the cells were stained with oil red O and imaged under a microscope.

### In Vivo Imaging

After culturing under PH or normoxia, SSCs were digested with trypsin, and single‐cell suspensions of ≈1 mL were formed. Then, 10 µL of DMSO‐dissolved DIR dye was added, and incubated for 10 min at 37 °C in the dark. Following centrifugation, the supernatant was discarded, and a complete culture medium was added to the SSC precipitate for one wash. After centrifugation again, the cell pellets were collected, ≈100 µL of complete medium was added to re‐suspend the cell precipitates, and cell counting was performed. Finally, the DIR‐labeled SSCs were injected into the IVDs, which were imaged in vivo at different time points.

### Live/Dead Staining

A Calcein‐AM/PI cell viability/cytotoxicity assay kit (cat. no. BL130A, Biosharp, Anhui, China) was used to distinguish live and dead cells. The cells were seeded on a 48‐well plate and incubated with Calcein‐AM (4 µm) and PI (6 µm) for 30 min at 37 °C. After washing with PBS, the cells were observed and photographed under a microscope.

### RT‐qPCR

Total RNA was isolated using RNAiso Plus reagent (cat.no. 9109; Takara Bio, Shiga, Japan). cDNA was synthesized from RNA using the PrimeScript RT Reagent Kit with gDNA Eraser (cat. no. RR047A; Takara Bio). RT‐qPCR was performed with TB Green *Premix Ex Taq* II (cat. no. RR820A; Takara) on a CFX Connec Real‐Time PCR Detection System (Bio‐Rad, Hercules, CA, USA). Primers were purchased from Tsingke Biotechnology (Chongqing, China).

### Western Blotting

Cells were lysed using radioimmunoprecipitation assay lysis buffer. Then, 5× sodium dodecyl sulfate‐polyacrylamide gel electrophoresis (SDS‐PAGE) sample loading buffer was added to the samples, and electrophoresis was performed at 120 V for 70–100 min. Next, the total protein was transferred to a polyvinylidene fluoride (PVDF) membrane at 300 mA for 90–120 min. The PVDF membrane was blocked and incubated with appropriately diluted primary antibodies at 4 °C overnight. After washing with Tris‐buffered saline containing 0.1% Tween 20 (TBST) buffer three times for 10 min each time, the PVDF membrane was incubated with a diluted secondary antibody at 25 °C for 1–2 h. After washing with TBST, signals on the PVDF membrane were detected using a chemiluminescence kit (cat. no. BG0001; BIOGROUND, Chongqing, China) on an imaging system (Bio‐Rad).

### Histology Assay

IVD tissues or renal specimens were fixed in 4% paraformaldehyde for 2–3 days. After ethylenediamine tetraacetic acid (EDTA) decalcification, tissue sections were stained with hematoxylin and eosin, Movat's pentachrome (cat. no. BP‐DL318; SenBeiJia, Nanjing, China), or safranin O‐fast green (cat. no. G1371; Solarbio).

### ROS assay

Cell or tissue ROS production was detected using the Tissue Section Reactive Oxygen Species Detection Kit (cat. no. BB‐470513; BestBio, Shanghai, China). Rat IVDs were embedded in an OCT compound to prepare frozen sections. Cell or tissue samples were washed for 5 min with cleaning solution B, 100–200 µL of dyeing solution A was added, and the samples were incubated for 20–60 min in the dark. After washing, samples were counterstained for 5 min with 4′,6‐diamidino‐2‐phenylindole and imaged under a microscope (Olympus).

### Flow Cytometry

To identify SSCs with stem cell characteristics, cell suspension was incubated with Tie2 and ENPEP antibodies at 37 °C for 1–2 h. After washing, cell suspension was incubated with Alexa Fluor 488‐conjugated anti‐rabbit IgG at 37 °C for 1–2 h. During the last 30 min of incubation, CD90, CD45, CD51, CD105, and OX‐83 antibodies and eBioscienc Fixable Viability Dye eFluor 780 were added to the cell suspension. CD90^−^CD45^−^CD105^−^ENPEP^−^Tie2^−^OX‐83^−^CD51^+^ SSCs were identified and sorted by flow cytometry. To measure and analyze the cell survival rate after the SSCs entered the IVDs, they were stained with Calcein‐AM and counted after being washed with PBS. ≈15 000 Calcein‐AM‐positive SSCs were injected into IVDs. After 2–3 days, all cells in the IVDs were obtained and then filtered with a 40 µm filter. After centrifugation, the cell pellets were obtained and resuspended in 500 µL PBS. Flow cytometry was used to collect 50 000 cells and then calculate the volume *V* of the remaining cell suspension. The number *N* of Calcein‐AM‐positive SSCs was calculated based on the positive ratio *R*. Thus, the number *N* of remaining Calcein‐AM‐positive SSCs in the IVD after 2–3 days was calculated as *N* = 50 000 × R × 500/(500–V).

### Hypoxic Culture of SSCs

Normoxic SSCs were cultured in an incubator at 21% O_2_. For hypoxia, SSCs were transitioned from 21% to 1% O_2_ for 2–3 days in a hypoxic incubator (Whitley H35 Hypoxystation; Don Whitley, Bingley, UK). For PH preconditioning, the SSCs were pre‐cultured under a gradually decreasing O_2_ concentration gradient (21%, 15%, 12%, 9%, 6%, 3%, 1%) for 2–3 days, reducing the O_2_ concentration approximately every 8–12 h. Then, the PH‐preconditioned SSCs were subjected to subsequent related experiments, such as IVD injection. Cell viability was determined using a Cell Counting Kit‐8 (cat. no. BG0025; BIOGROUND, Chongqing, China).

### Lipid Peroxidation Assay

C11‐BODIPY 581/591 (cat. no. D3861; Thermo Fisher Scientific) or a CellROX Deep Red Flow Cytometry Assay Kit (cat. no. C10491, Thermo Fisher Scientific) was used to analyze ROS and lipid peroxidation levels. The treated cells were incubated with C11‐BODIPY or CellROX Deep Red reagent for 30 to 60 min at 37 °C. The cells were then washed three times with PBS and analyzed using flow cytometry. For cells stained with the CellROX Deep Red reagent, fluorescence emission was collected with 665/40 BP filters. Cells were stained with C11‐BODIPY using the traditional Texas Red (590 nm) and fluorescein isothiocyanate (510 nm) emission filters.

### GSH Assay

GSH colorimetric assay kit (cat. no. E‐BC‐K030‐M; Elabscience, Wuhan, China) was used to measure the GSH levels. The cells were collected, washed twice before sonication in an ice‐water bath, and centrifuged at 1500 × *g* for 10 min. The supernatant was incubated with 100 µL of acid reagent at room temperature and then centrifuged for 10  min at 4500 × *g*. The protein concentration in the supernatant was measured using a BCA kit. Twenty‐five microliters of DTNB solution and 100 µL of the resulting supernatants were added to a 96‐well plate and incubated with 100 µL of phosphate for 5 min at room temperature. The absorbance of each well was measured at 405 nm using a microplate reader. The amount of GSH in the cells was calculated based on a GSH standard curve and normalized to the protein concentration.

### MDA Assay

MDA levels in the cells were measured using an MDA colorimetric assay kit (cat. no. E‐BC‐K028‐M; Elabscience). Briefly, the cells were collected and sonicated in the extraction solution for 10 min. Samples (100 µL) were incubated with 1000 µL of working solution at 100 °C for 40 min and then centrifuged at 1078 × *g* for 10 min. The absorbance of the supernatant (250 µL) was measured at 532 nm on a microplate reader. The protein concentration in the supernatant was measured using a BCA kit. The amount of MDA in each sample was normalized to the protein concentration.

### Iron Assay

Intracellular iron levels were detected using an iron colorimetric assay kit (cat. no. E1042; Applygen, Beijing, China). In brief, cells were harvested, washed three times, and then lysed for 2 h. Samples (100 µL) were incubated with 100 µL working solution at 60 °C for 60  min. Then, 30 µL of iron detection agent was added, and the mixture was incubated for 30 min. The absorbance of the supernatant (200 µL) was measured at 550 nm on a microplate reader. An iron standard curve was prepared, and the amount of iron was normalized to the protein content.

### Lentivirus and si‐RNA Transfection

Lentiviruses to overexpress *foxo3* and *hif‐1α* and si‐RNA to silence FOXO3 were obtained from GenePharma (Shanghai, China). When the SSCs reached 50% confluence, they were transfected with lentivirus or si‐RNA according to the manufacturer's instructions. We used western blotting to analyze the transfection efficiency.

### Obtained the SSCs After Injected Into IVD

SSCs were injected into IVDs after being cultured under normoxia or PH preconditioning. After two to three days, SSCs were not fully integrated with the surrounding nucleus pulposus tissue, which still existed as a single cell in the nucleus pulposus region. At this time, the rats were euthanized, the IVDs were opened, and the tissues and cells in the nucleus pulposus region were gently dissociated with PBS and collected in PBS solution. After gently shaking and filtration with a 40 µm filter, a single SSC suspension was obtained. Cell precipitate containing a large number of SSCs was obtained by centrifugation, and then flow cytometry, western blotting, and transmission electron microscopy (TEM) were performed.

### Synthesis and Characterization of PDA‐NPs and C‐176@PDA‐NPs

3,4‐Dihydroxyphenethylamine hydrochloride (dopamine hydrochloride) (0.1 g, CAS: 62‐31‐7; KAIWEI CHEMICAL, Shanghai, China) was weighed, and 110 mL of deionized water and 40 mL of ethanol were added. A 1 M NaOH solution was used to adjust the pH to ≈9–10. After stirring at room temperature for 5–6 h and centrifuging at 10 000 rpm for 10 min, the precipitated dopamine nanoparticles (PDA‐NPs) were collected. For the preparation of C‐176@PDA‐NPs, C‐176 was dissolved in ethanol. Then, 0.1 g of dopamine hydrochloride was weighed and dissolved in 5.5 mL of deionized water and 2 mL of ethanol. The C‐176 solution was added to the dopamine solution, and 1 M NaOH solution was used to adjust the pH to ≈9–10. The resulting solution was stirred overnight. Subsequently, the precipitate of PDA‐NPs coated with C‐176 (C‐176@PDA‐NPs) was collected through centrifugation at 10 000 rpm for 10 min. The elements in PDA‐NPs and C‐176@PDA‐NPs were characterized using HAADF‐STEM and EDS in Talos F200X Transmission Electron Microscopy (Thermo Fisher Scientific, USA).

### Characterization of C‐176 Released from C‐176@PDA‐NPs

C‐176@PDA‐NPs (5–10 mg) were dissolved in deionized water (1 mL) and added to a dialysis bag after complete dissolution. The release medium was 10 mL of 25% ethanol solution. The C‐176@PDA‐NPs solution was shaken at 37 °C with a rotating speed of 200 rpm. Samples of 200–400 µL were obtained at 12, 24, 48, 72, 120 and 168 h, and an equal volume of fresh release medium at the same temperature was added back to the dialysis bag. The absorbance value A at the corresponding time point was measured at a wavelength of 550 nm using a Tecan Infinite 200 microplate reader (Tecan, Morrisville, NC, USA). C‐176@PDA‐NPs (5–10 mg) were dissolved in NaOH solution to disrupt the nanoparticles and release C‐176. The release medium was 10 mL of a 25% ethanol solution. The C‐176@PDA‐NPs solution was shaken at 37 °C with a rotating speed of 200 rpm. Samples of 200–400 µL were taken after C‐176 was evenly distributed in the release medium, and the absorbance value B was measured at a wavelength of 550 nm by the microplate reader. Based on an absorbance standard curve, the concentration of C‐176 corresponding to absorbance values A or B was calculated to determine the cumulative release rate of C‐176.

### Synthesis and Characterization of HAMA and HAMA‐C‐176@PDA‐NPs Hydrogels

Hyaluronic acid (0.5 g, cat. no. H909938‐25g; MACKLIN, Shanghai, China) was added to 50 mL deionized water and fully dissolved. Then, 2 mL of methacrylic anhydride (cat. no. RH38536‐25ml; BioRuler, Beijing, China) was added. Next, 4 M NaOH was continuously added to maintain a PH value of 8–9, and the solution was stored at 4 °C overnight. Starting the next day, dialysis was performed in deionized water for 3 days using a dialysis bag with an intercepted molecular weight of 8000–14 000 Dalton. HAMA solids were obtained by freeze‐drying. After dissolution in a solution containing photoinitiator I2959, the HAMA solution quickly formed a hydrogel under UV light. HAMA‐C‐176@PDA‐NPs hydrogels were prepared by cross‐linking C‐176@PDA‐NPs with HAMA using 3‐APBA. First, 3‐APBA was added to the HAMA solution, and N‐hydroxysuccinimide (NHS) and 1‐ethyl‐3‐(3‐dimethylaminopropyl) carbodiimide (EDC) hydrochloride were added to catalyze the formation of an amide bond between the ‐COOH in HAMA and ‐NH_2_ in 3‐APBA. Then, C‐176@PDA‐NPs were added to form borate ester bonds at room temperature for ≈4 h so that the C‐176@PDA‐NPs were connected to 3‐APBA. Finally, the unreacted 3‐APBA and excess C‐176@PDA‐NPs were removed by centrifugation and dialysis to obtain the HAMA‐C‐176@PDA‐NPs solution. HAMA‐C‐176@PDA‐NPs solids were obtained after freeze‐drying. After dissolution in a solution containing the photoinitiator I2959, the HAMA‐C‐176@PDA‐NPs solution quickly formed hydrogels under UV light. To improve cell growth, a small amount of collagen type I protein was added when SSCs were cultured in HAMA or HAMA‐C‐176@PDA‐NPs hydrogels.

### Characterization of PDA‐NPs Released from HAMA‐PDA‐NPs

Take an appropriate amount of PDA‐NPs, add 1 mL of ddH2O, mix well, and then add it to the HAMA solution to be photocured into a hydrogel. Use 20 mL of ddH_2_O as the release medium. During the release process of HAMA‐PDA‐NPs in ddH_2_O, at 0, 12, 24, 48, 72, 120 and 168 h, take 200–400 µL of the release medium and measure the absorbance value (A). For a control, take an equal amount of PDA‐NPs, add 1 mL of ddH_2_O, mix well, and then add it to 20 mL of ddH_2_O. Measure the absorbance value (B) at 0, 12, 24, 48, 72, 120 and 168 h. Then calculate the release rate of PDA‐NPs according to the standard curve of PDA‐NPs.

### Animal Experiments

Sprague–Dawley rats were purchased from the Experimental Animal Center of the Army Military Medical University (Chongqing, China). Rats were anesthetized with Delivector Avertin (10–20 µL g^−1^). SSCs were extracted from 2‐week‐old rats. Six 4‐week‐old rats were used for kidney subcapsular transplantation of SSCs.

Twelve rats (≈200 g) were evenly divided into four groups: NC, puncture, puncture + SSCs, and puncture + SSCs treated with PH. A 21 G needle was used to puncture the annulus fibrosus of the rats. Then, the needle was rotated 360° and maintained in the disc for 60 s. Rats were subjected to the intradiscal injection of SSCs treated with or without PH preconditioning. The total volume was ≈20 µL, containing ≈20–40 × 10^4^ SSCs. X‐ray, magnetic resonance imaging, and safranin O‐fast Green staining were then performed.

Six 2‐month‐old rats (≈200 g) were evenly divided into two groups for in vivo imaging after the IVDs were injected with DIR‐labeled SSCs or PH‐pretreated DIR‐labeled SSCs. Ten 2‐month‐old rats (≈200 g) were divided into two groups to determine the survival of SSCs in the IVDs. Five or six IVDs in each rat were injected with 15 000 Calcein‐AM^+^ SSCs or PH‐preconditioned Calcein‐AM^+^ SSCs. Nine 2‐month‐old rats were evenly divided into three groups for ROS staining after the IVDs were injected with PBS, SSCs or PH‐preconditioned SSCs.

Nine rats were evenly divided into three groups to assess the toxicity of the hydrogel at 1, 4, and 8 weeks. Thirty‐six rats were evenly divided into six groups: NC, puncture, puncture + HAMA hydrogel, puncture + HAMA‐C‐176@PDA‐NPs, puncture + HAMA hydrogel + SSCs, and puncture + HAMA hydrogel + C‐176@PDA‐NPs + SSCs. In the first week, the IVDs in eighteen of the rats were stained with ROS. The remaining eighteen rats underwent MRI, X‐ray, and tissue staining at week 8. For micro‐MRI or X‐ray analysis, a 7.0 T animal magnet (Bruker Pharmascan, Ettlingen, Germany) or an AniView100 multi‐mode animal live imaging system (BLT, Guangzhou, China) was used to evaluate rat IVD signals and structural changes. The animal use and experimental procedures met the requirements of the Guidelines for the Care and Use of Laboratory Animals of the National Institutes of Health and were approved by the Animal Ethics Committee of the Army Medical University (no. AMUWEC20230455).

### Statistical Analysis

All data were presented as mean ± standard deviation. The data were analyzed using the Student's *t*‐test or one‐way analysis of variance followed by Tukey's test in GraphPad Prism 7.0 (GraphPad Software Inc., CA, USA). Statistical significance was set at *p* < 0.05.

## Conflict of Interest

The authors declare no conflict of interest.

## Supporting information



Supporting Information

## Data Availability

The data that support the findings of this study are available from the corresponding author upon reasonable request.
