# Supplementary material for: 3‐D Sustained‐Release Culture Carrier Alleviates Rat Intervertebral Disc Degeneration by Targeting STING in Transplanted Skeletal Stem Cells
Source: Adv Sci (Weinh). 2025 Feb 22;12(15):2410151. doi: 10.1002/advs.202410151 (PMC12005824; doi:10.1002/advs.202410151)
Supplement: Supplementary file 1 — Supporting Information [file ADVS-12-2410151-s001.docx]

Supplementary Materials for

3-D sustained-release culture carrier alleviates rat intervertebral disc degeneration through targeting STING in transplanted skeletal stem cells

Liwen Luo *et al.*

*Corresponding author: Yao Liu, liuyao@tmmu.edu.cn. Bing Ni, nibing@tmmu.edu.cn. Changqing Li, changqli@tmmu.edu.cn. Zhiqiang Tian, tzhiq009@ tmmu.edu.cn.

**
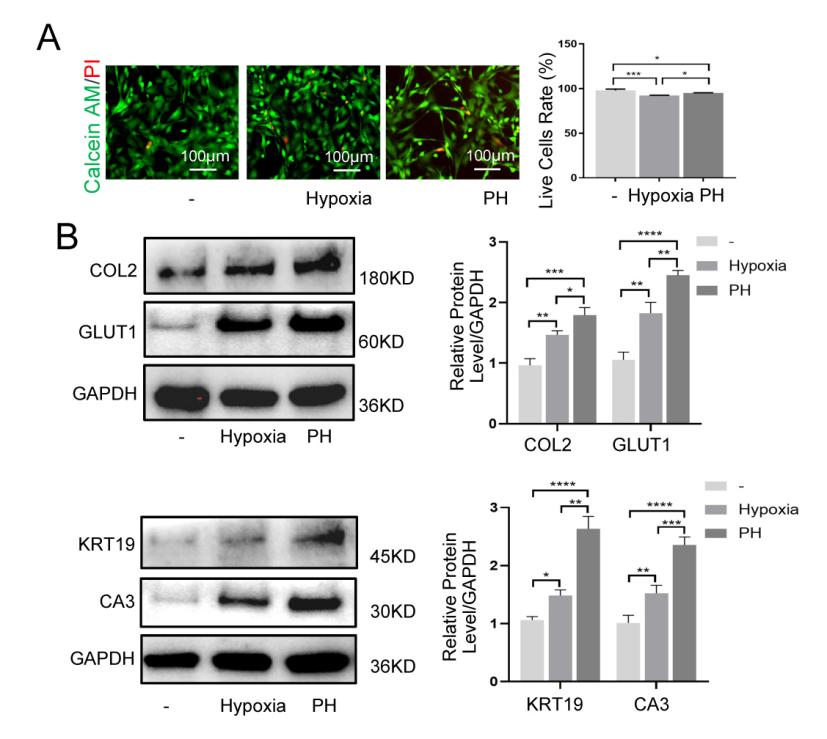
**

**Figure S1. PH preconditioning contributed to SSCs adapting to the hypoxic**

**environment. A**) Cell live/dead staining and statistical analysis of SSCs treated with normoxia, hypoxia, or PH. **B**) Western blotting and quantitative protein levels of COL2, GLUT1, KRT19, and CA3 in SSCs treated with normoxia, hypoxia, or PH. SSCs, skeletal stem cells; COL2, collagen II; GLUT1, glucose transporter-1; KRT19, keratin 19; CA3, carbonic anhydrase 3. Data in (**A, B**) are presented as the mean ± SD, *p < 0.05, **p < 0.01, ***p < 0.001, ****p < 0.0001, ns, no significant difference by one-way analysis of variance (ANOVA).


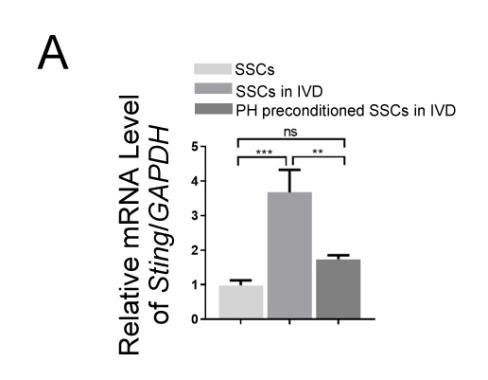


Figure S2. Detection of STING expression in SSCs entering IVDs. The mRNA level of STING in SSCs cultured under normoxia *in* *vitro*, normoxic or PH-preconditioned SSCs entering the IVD. Data are presented as the mean ± SD; **p < 0.01, ***p < 0.001, ns, no significant difference by one-way analysis of variance (ANOVA) test.


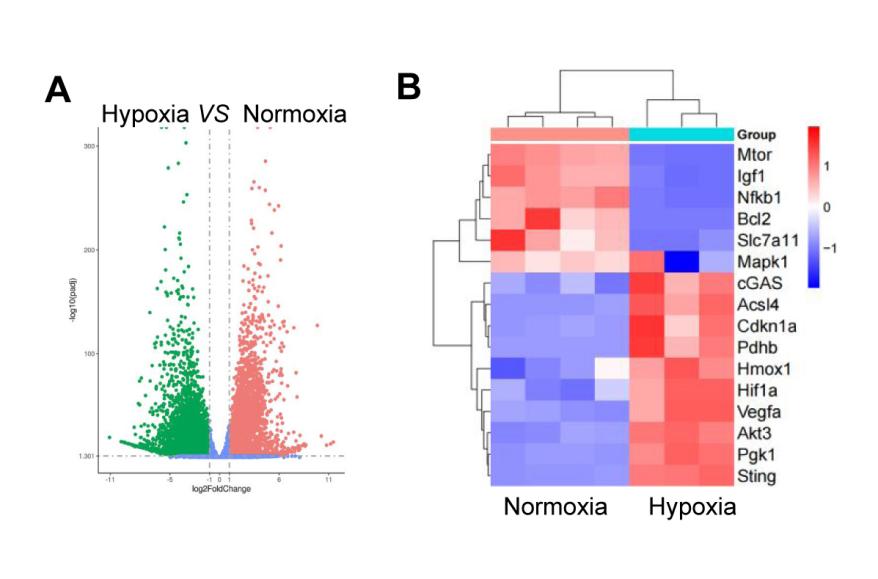


**Figure S3**. Analysis of RNA-seq results in SSCs treated with normoxia or hypoxia. Volcano plots (**A**) and heat map (**B**) showing the differences in gene expression between normoxic and hypoxic SSCs.


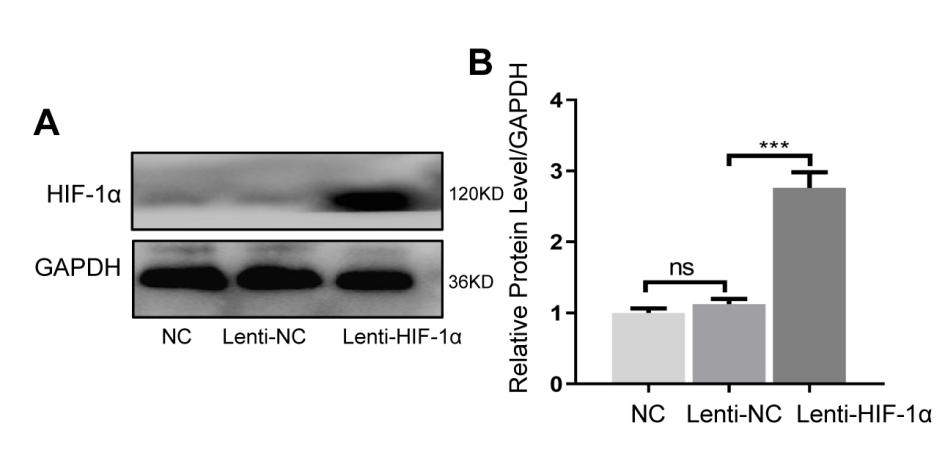


**Figure S4.** Western blotting (**A**) and quantitative protein levels (**B**) of HIF-1α in the NC, Lenti-NC, and Lenti-HIF-1α groups. Data in (**B**) are presented as the mean ± SD, ***p < 0.001, ns, no significant difference by one-way ANOVA.


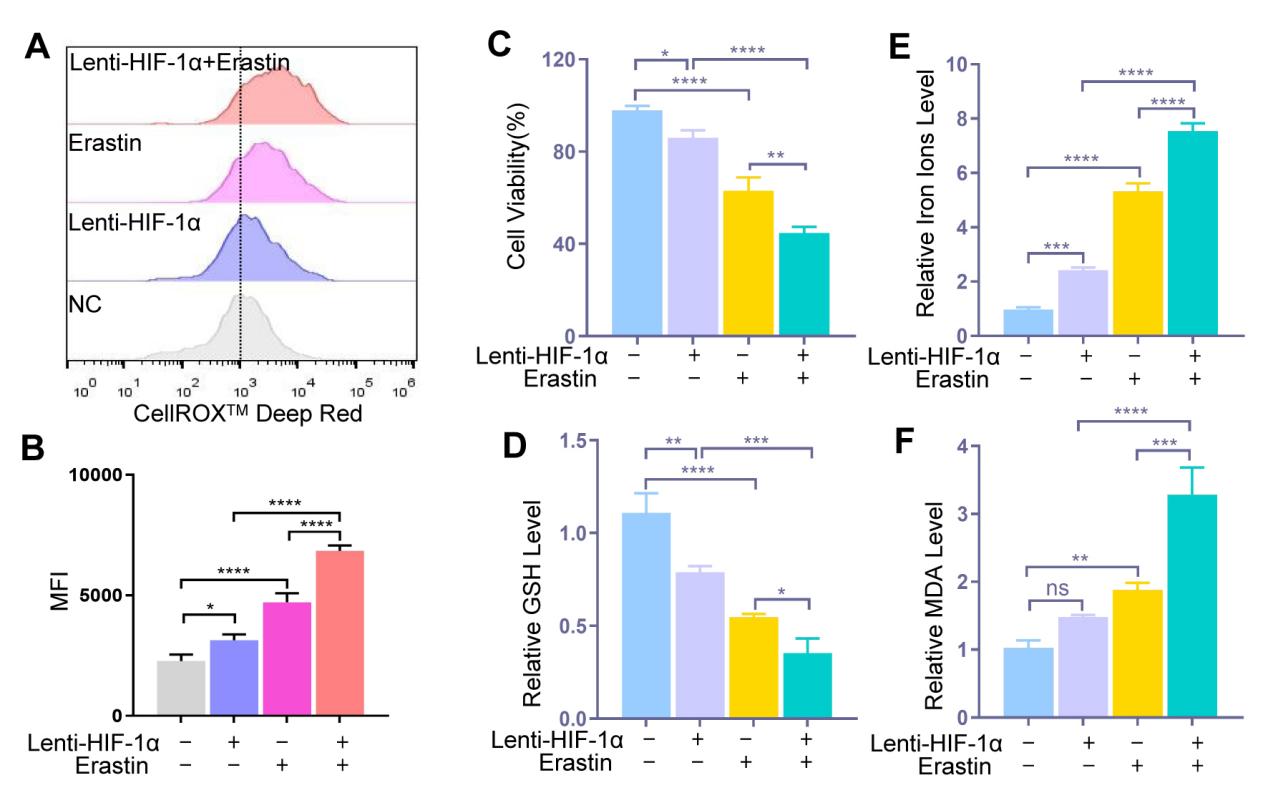


**Figure S5. HIF-1α enhances ferroptosis in SSCs.** **A, B**) CellROX™ Deep Red staining for oxidative stress and quantitative mean fluorescence intensity (MFI) in SSCs in the NC, Lenti-HIF-1α, erastin (10 μM), and Lenti-HIF-1α + erastin (10 μM) groups. (**C–F**) Cell viability, GSH level, intracellular iron ion concentration and MDA level in SSCs in the NC, Lenti-HIF-1α, erastin, and Lenti-HIF-1α + erastin groups. Data in (**B–F**) are presented as the mean ± SD, *p < 0.05, **p < 0.01, ***p < 0.001, ****p < 0.0001, ns, no significant difference by one-way ANOVA.


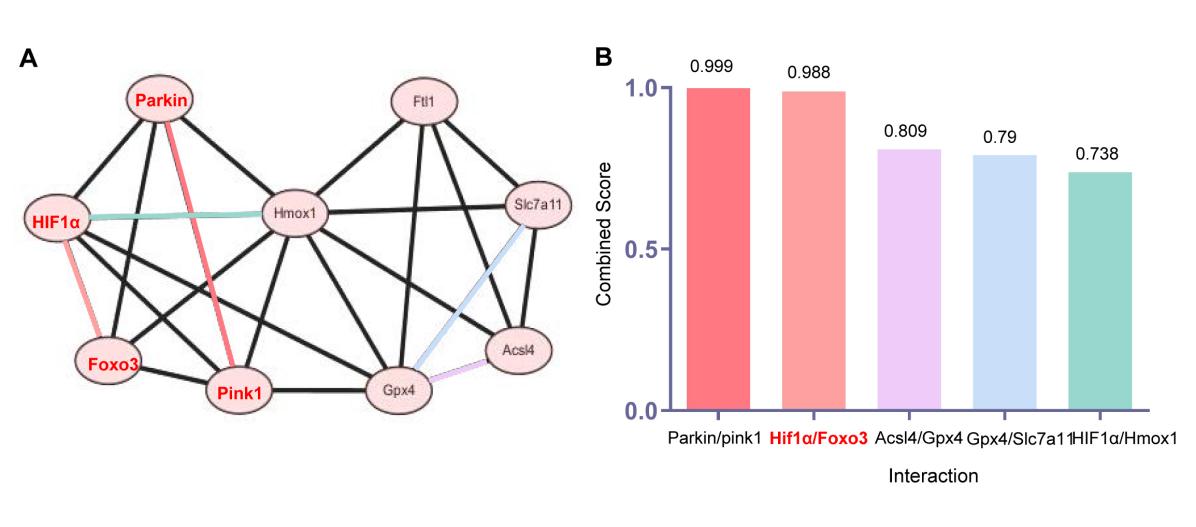


**Figure S6. Protein interactions among HIF-1α, FOXO3, and mitophagy- and ferroptosis-related proteins**. **A**) Protein interaction network of HIF-1α, FOXO3, PINK1/PARKIN, and GPX4/ACSL4/HMOX1/SLC7A11/FTL1 via STRING database and Cytoscape software. **B**) Combined score analysis of HIF-1α, FOXO3, and mitophagy- and ferroptosis-related genes. Abbreviations: HIF-1α, hypoxia-inducible factor-1 alpha; FOXO3, forkhead box O3; HMOX-1, heme oxygenase 1; SLC7A11, solute carrier family 7 member 11; FTL1, ferritin light chain 1.


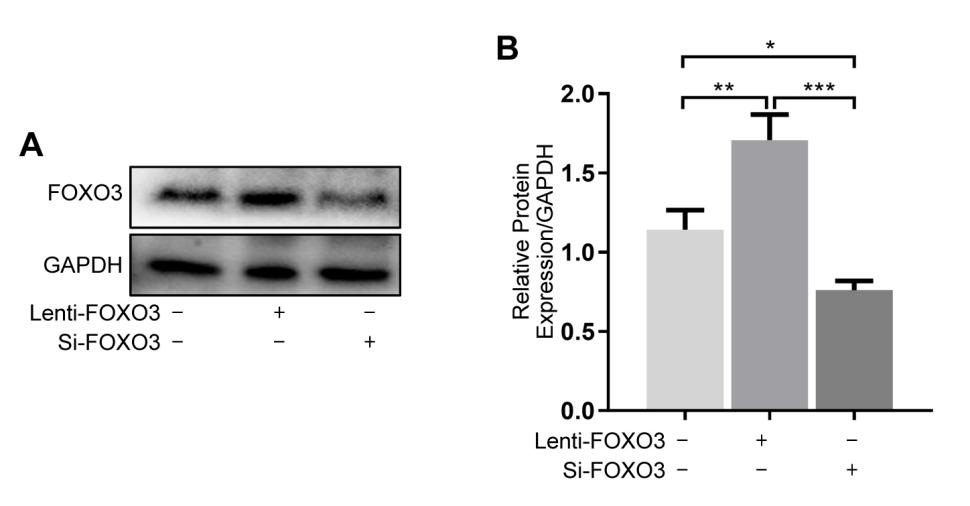


**Figure S7. Expression of FOXO3 in SSCs is increased by Lenti-FOXO3 and decreased by si-FOXO3, respectively.** **A, B**) Western blotting and quantitative protein levels of FOXO3 in SSCs in the NC, Lenti-FOXO3, and si-FOXO3 groups. Data in (**B**) are presented as the mean ± SD, *p < 0.05, **p < 0.01, ***p < 0.001 by one-way ANOVA.


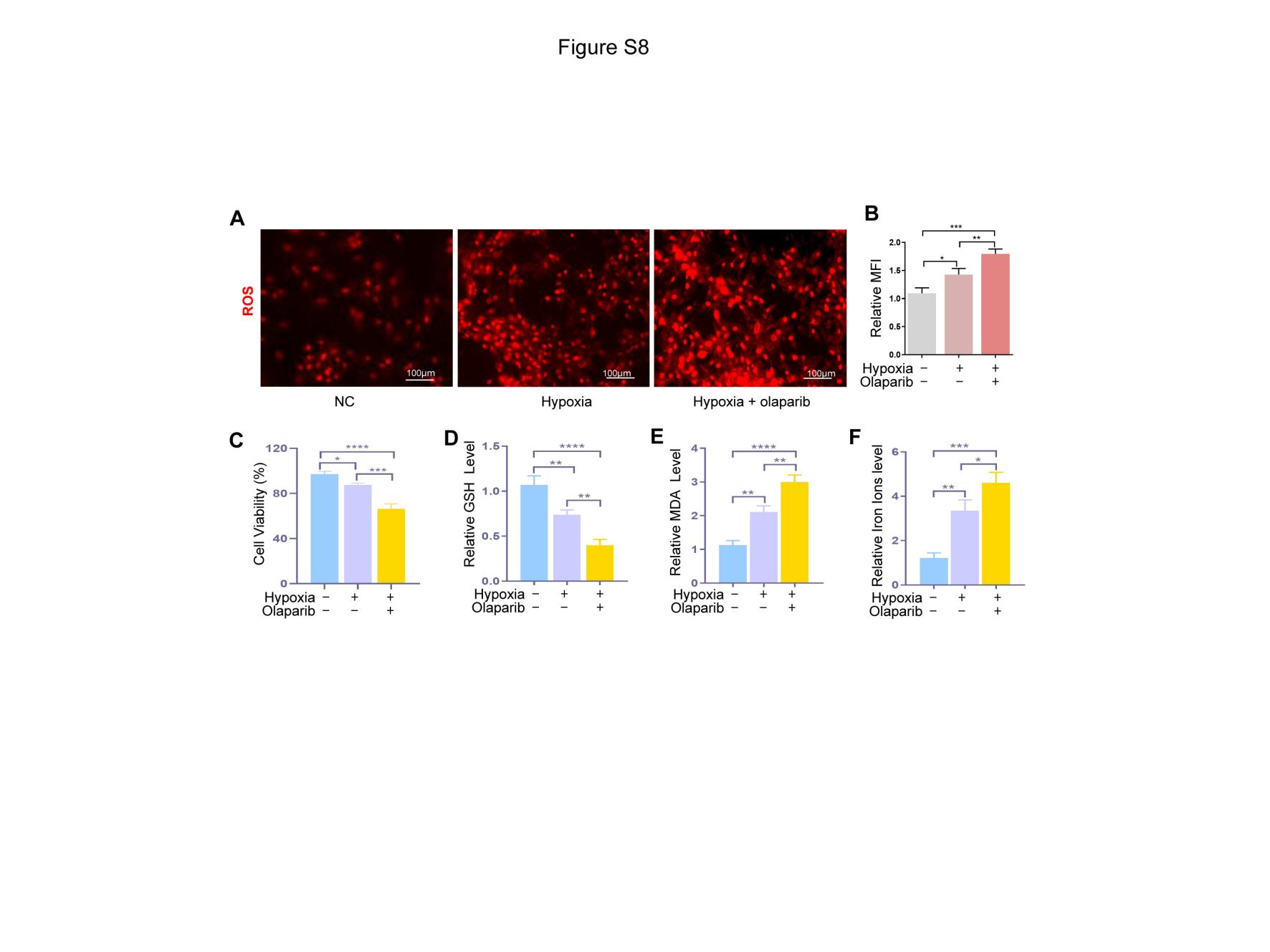


**Figure S8. Mitophagy aggravates ferroptosis in SSCs. A, B**) ROS staining and quantitative MFI of SSCs in the NC, hypoxia, and hypoxia + olaparib groups. **C–F**) Cell viability, GSH, MDA, and intracellular iron ion levels in SSCs in the NC, hypoxia, and hypoxia + olaparib groups. Data in (**B–F**) are presented as the mean ± SD, *p < 0.05, **p < 0.01, ***p < 0.001, ****p < 0.0001, ns, no significant difference by one-way ANOVA followed by Tukey’s test.


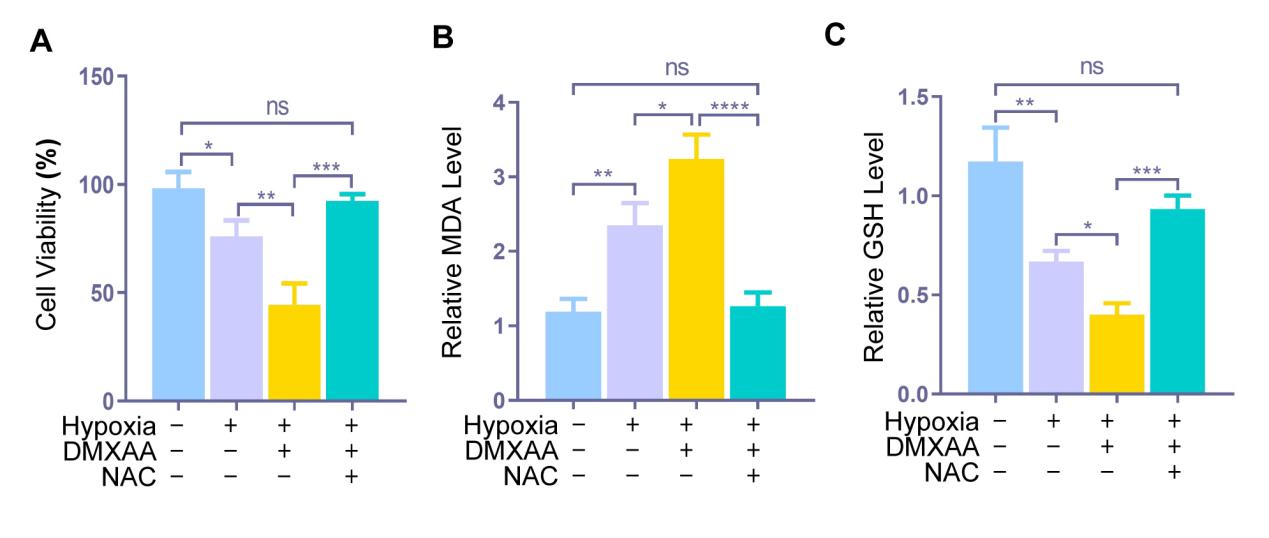


**Figure S9.** **A–C)** Cell viability and MDA and GSH levels detected in SSCs treated with NC, hypoxia, hypoxia + DMXAA, and hypoxia + DMXAA + NAC. Data in (**A–C**) are presented as the mean ± SD, *p < 0.05, **p < 0.01,***p < 0.001, ****p < 0.0001, ns, no significant difference by one-way ANOVA.


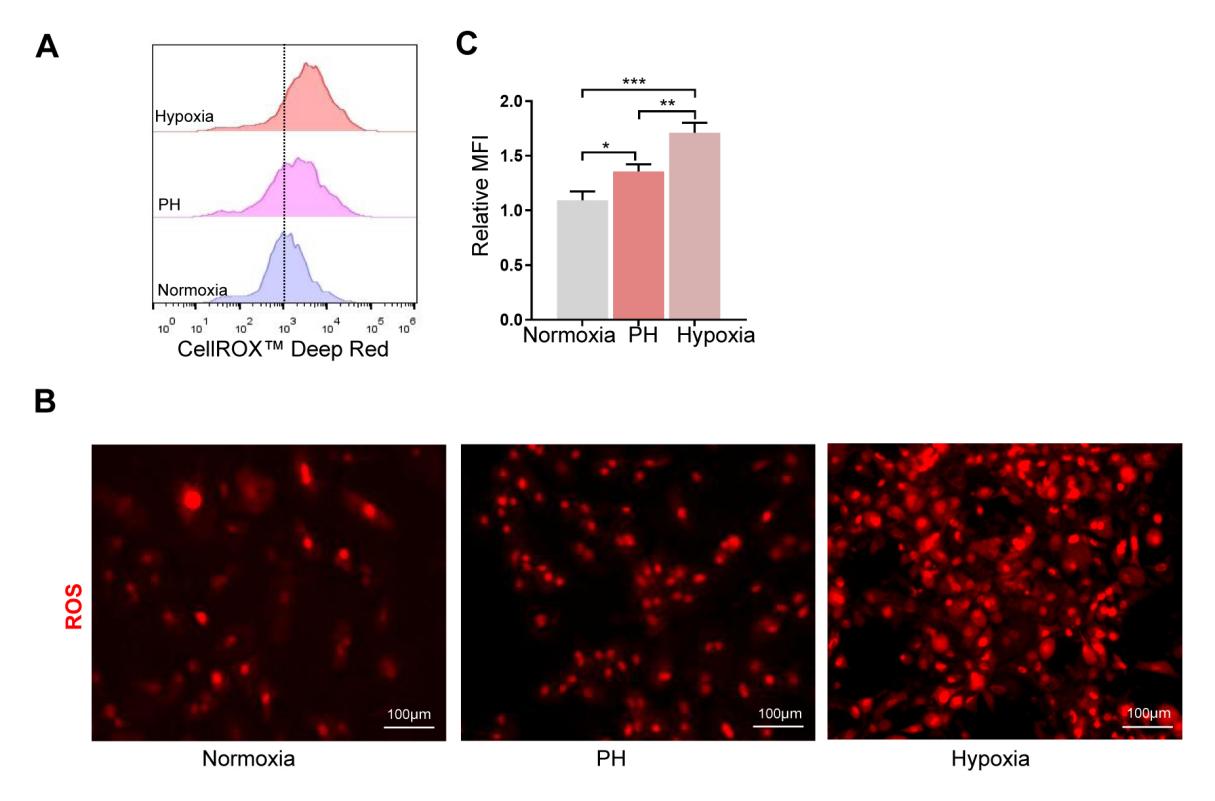


**Figure S10. PH preconditioning attenuates SSCs ferroptosis. A**) CellROX^TM^ Deep Red staining and statistical analysis of MFI to detect lipid peroxidation in SSCs in the NC, hypoxia, and PH groups. **B, C**) ROS staining and quantitative MFI analysis in SSCs in the NC, hypoxia, and PH groups. Data in (**C**) are presented as the mean ± SD, *p < 0.05, **p < 0.01, ***p < 0.001 by one-way ANOVA.


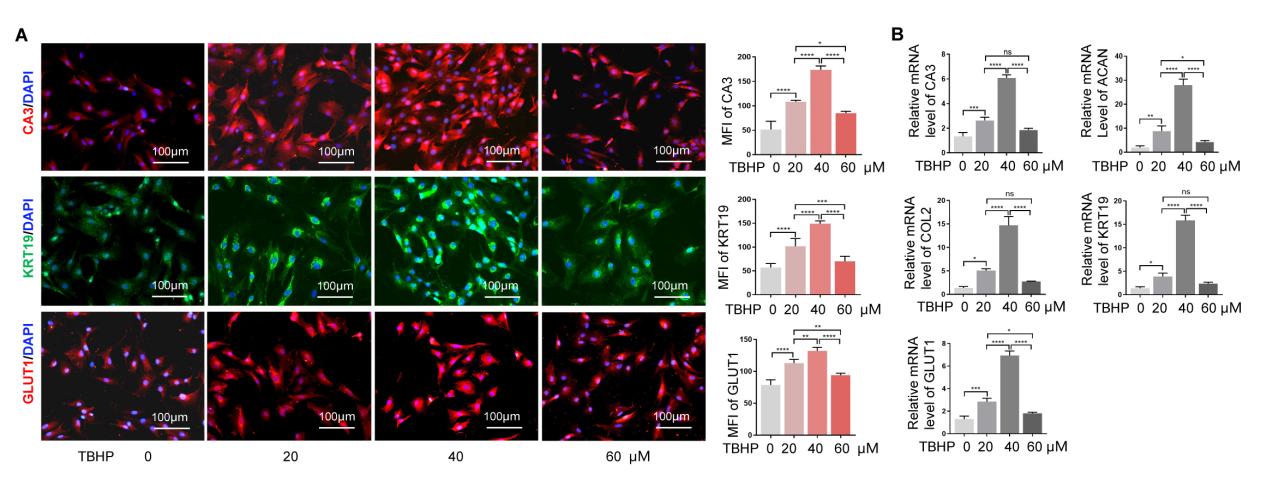


**Figure S11. ROS promotes the differentiation of SSCs into NPLCs.** **A**) Immunofluorescence staining and MFI of CA3, KRT19 and GLUT1 in SSCs treated with TBHP at concentrations of 0, 20, 40 or 60 μM. B) The mRNA expression level in SSCs treated as above. Data in (A-B) are presented as the mean ± SD, *p < 0.05, **p < 0.01, ***p < 0.001, ****p < 0.0001, ns, no significant difference by one-way ANOVA followed by Tukey’s test.


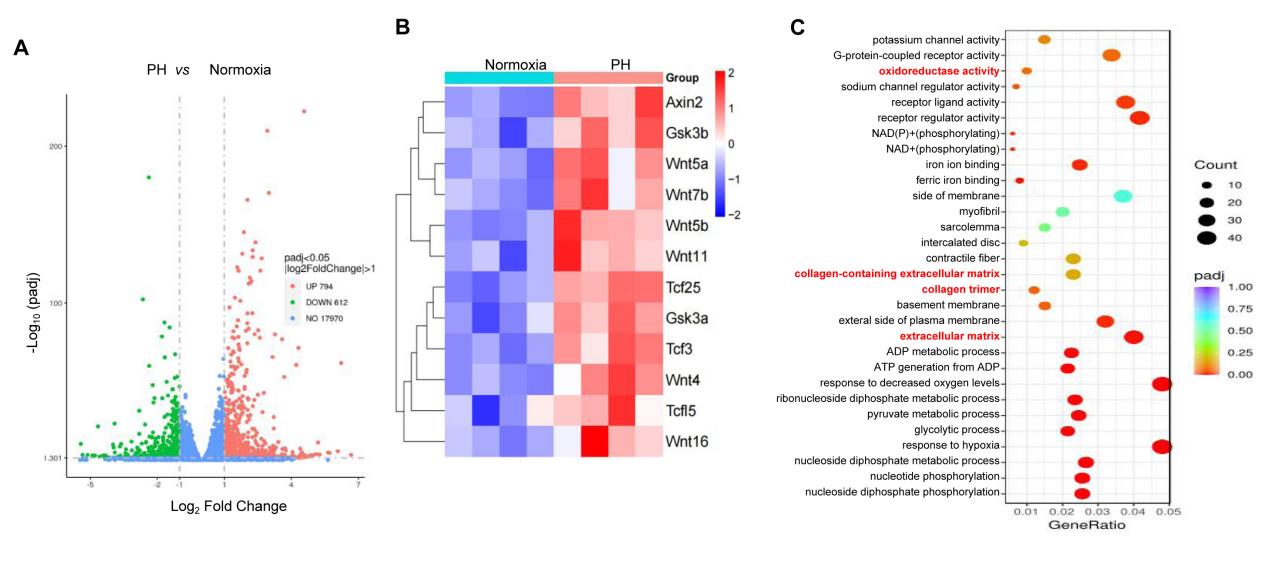


**Figure S12. Analysis of RNA-seq results. A**) Volcano plot of gene expression in SSCs cultured under normoxia and PH. **B**) Heatmap analysis of Wnt pathway-related genes in SSCs cultured under normoxia and PH. **C**) KEGG pathway analysis of SSCs cultured under normoxia and PH.


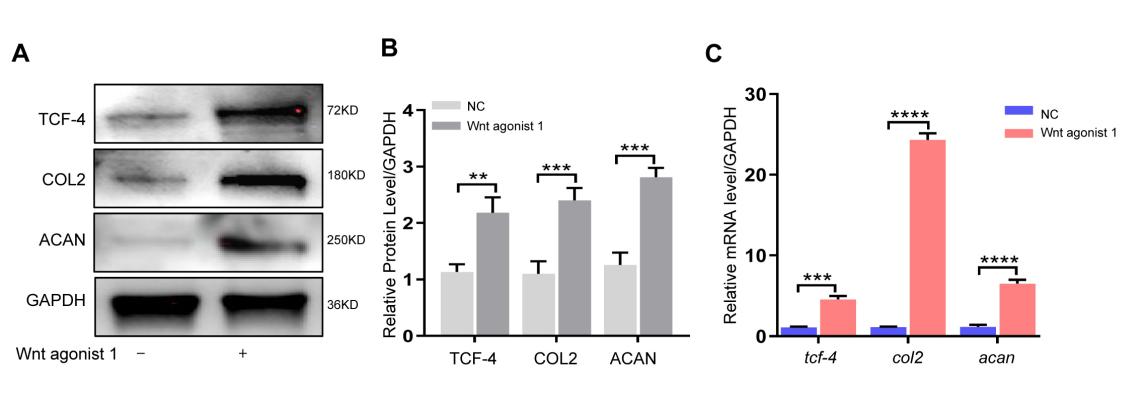


**Figure S13. Activation of the Wnt pathway promotes the differentiation of SSCs into NPLCs. A, B**) Western blotting and quantitative protein levels of TCF-4, COL2, and ACAN in SSCs treated with Wnt agonist 1 (10 μM) compared to untreated SSCs. **C**) mRNA levels of TCF-4, COL2, and ACAN in SSCs treated with Wnt agonist 1 (10 μM) compared to untreated SSCs. Data in (**B, C**) are presented as the mean ± SD, ***p < 0.001, ****p < 0.0001 by two-tailed Student’s t-test.


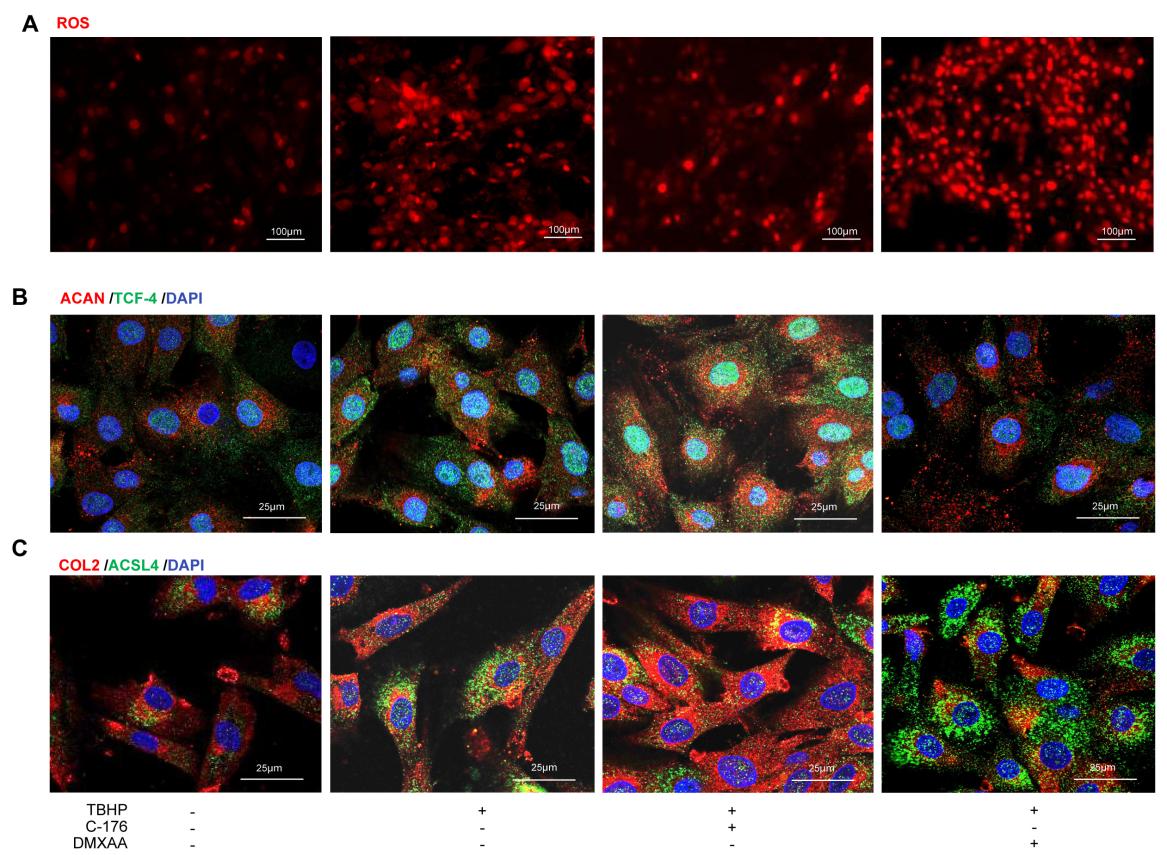


**Figure S14. STING regulates the balance of ferroptosis and differentiation in SSCs by influencing ROS production. A)** ROS staining of SSCs in the NC, TBHP, TBHP + C-176, and TBHP + DMXAA groups. **B, C**) Immunofluorescence staining of ACAN (red)/TCF-4 (green) and COL2 (red)/ACSL4 (green) in SSCs in the NC, TBHP, TBHP + C-176, and TBHP + DMXAA groups. Abbreviations: STING, stimulator of interferon genes; ROS, reactive oxygen species; NC, normal control; TBHP, tert-butyl hydroperoxide; DMXAA, 5,6-dimethylxanthenone-4-acetic acid.


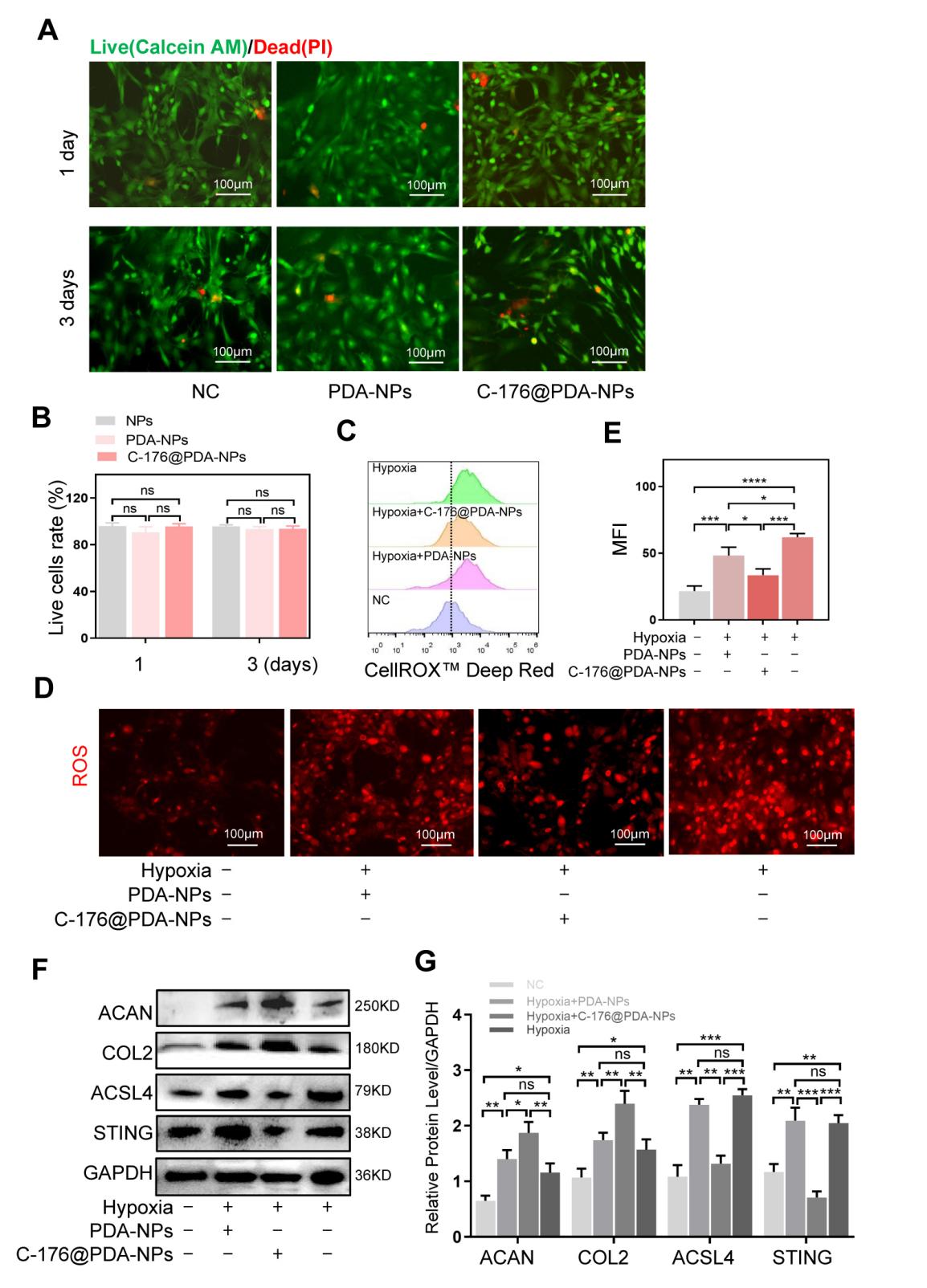


**Figure S15. C-176@PDA-NPs inhibit ferroptosis and promote the differentiation of SSCs by targeting STING.** **A, B**) Cell live/dead staining and statistical analysis of SSCs in the NC, PDA-NPs, and C-176@PDA-NPs groups. **C**) CellROX^TM^ Deep Red staining to detect lipid peroxidation in SSCs in the NC, PDA-NPs, and C-176@PDA-NPs groups. **D, E**) ROS staining and quantitative MFI in SSCs. **F, G**) Western blotting and quantitative protein levels of ACSL4, STING, COL2, and ACAN in SSCs in the NC, hypoxia + DPA-NPs, hypoxia + C-176@DPA-NPs, and hypoxia groups. Data in (**E, G**) are presented as the mean ± SD, *p < 0.05, **p < 0.01, ***p < 0.001; ns, no significant difference by one-way ANOVA.


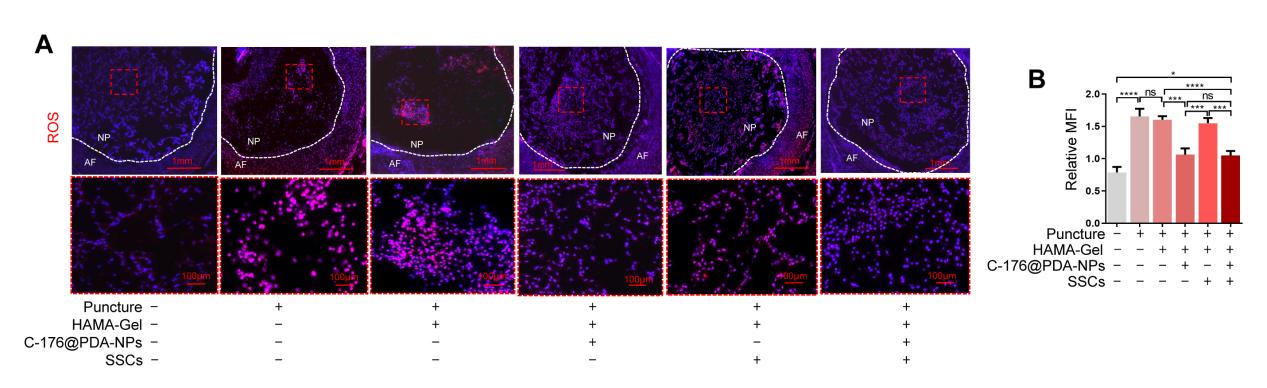


**Figure S16.** ROS staining (**A**) and quantified MFI (**B**) in rat IVDs treated with NC, puncture, puncture + HAMA hydrogel, puncture + HAMA-C-176@PDA-NPs, puncture + HAMA hydrogel + SSCs, and puncture + HAMA-C-176@PDA-NPs hydrogel + SSCs. Data in (**B**) are presented as the mean ± SD; *p < 0.05, **p < 0.01, ***p < 0.001, ****p < 0.0001, ns, no significant difference by one-way ANOVA.
